# Toward Heisenberg scaling in non-Hermitian metrology at the quantum regime

**DOI:** 10.1126/sciadv.adk7616

**Published:** 2024-05-10

**Authors:** Xinglei Yu, Xinzhi Zhao, Liangsheng Li, Xiao-Min Hu, Xiangmei Duan, Haidong Yuan, Chengjie Zhang

**Affiliations:** ^1^ School of Physical Science and Technology, Ningbo University, Ningbo 315211, China.; ^2^ National Key Laboratory of Scattering and Radiation, Beijing 100854, China.; ^3^ CAS Key Laboratory of Quantum Information, University of Science and Technology of China, Hefei 230026, China.; ^4^ CAS Center for Excellence in Quantum Information and Quantum Physics, University of Science and Technology of China, Hefei 230026, China.; ^5^ Department of Mechanical and Automation Engineering, The Chinese University of Hong Kong, Shatin, Hong Kong.; ^6^ Hefei National Laboratory, University of Science and Technology of China, Hefei 230088, China.

## Abstract

Non-Hermitian quantum metrology, an emerging field at the intersection of quantum estimation and non-Hermitian physics, holds promise for revolutionizing precision measurement. Here, we present a comprehensive investigation of non-Hermitian quantum parameter estimation in the quantum regime, with a special focus on achieving Heisenberg scaling. We introduce a concise expression for the quantum Fisher information (QFI) that applies to general non-Hermitian Hamiltonians, enabling the analysis of estimation precision in these systems. Our findings unveil the remarkable potential of non-Hermitian systems to attain the Heisenberg scaling of 1/*t*, where *t* represents time. Moreover, we derive optimal measurement conditions based on the proposed QFI expression, demonstrating the attainment of the quantum Cramér-Rao bound. By constructing non-unitary evolutions governed by two non-Hermitian Hamiltonians, one with parity-time symmetry and the other without specific symmetries, we experimentally validate our theoretical analysis. The experimental results affirm the realization of Heisenberg scaling in estimation precision, marking a substantial milestone in non-Hermitian quantum metrology.

## Teaser

The Heisenberg scaling in quantum non-Hermitian metrology is investigated in both theoretical and experimental ways.

## INTRODUCTION

The assumption of Hamiltonian Hermiticity has long been regarded as a fundamental requirement in quantum mechanics, ensuring real energy eigenvalues and unitary evolution. However, the emergence of research on non-Hermitian Hamiltonians with parity-time (PT) symmetry has challenged this concept and sparked substantial interest. This class of non-Hermitian Hamiltonians exhibits an intriguing property—an entirely real spectrum, especially at exceptional points (EPs), which serve as critical thresholds distinguishing the PT symmetry–broken and unbroken regimes (*1*, *2*). Over past two decades, there has been plenty of research and developments in various fields based on the interesting features of PT-symmetric non-Hermitian Hamiltonians, such as single-mode lasers (*3*–*5*), laser absorbers (*6*–*8*), topological mode transfer (*9*, *10*), metamaterials (*11*–*14*), and nonreciprocal device (*15*, *16*). These endeavors have reshaped our understanding and opened up frontiers in quantum science and engineering, leveraging the unique characteristics of non-Hermitian systems with PT symmetry.

Among various applications, non-Hermitian metrology has emerged as a captivating area of study, attracting considerable interest and attention. Previous studies have focused on achieving enhanced sensitivity near EPs in classical wave systems with balanced gain and loss (*17*–*21*). In addition, quantum noise theory of non-Hermitian sensors (*22*, *23*) and quantum Fisher information (QFI) in open systems (*24*, *25*) have been investigated. However, most of the implemented and discussed non-Hermitian systems in these works are still in the classical regime, the investigation of non-Hermitian quantum metrology in the quantum regime is still in its early stage.

Recent advancements have showcased promising progress in realizing non-Hermitian quantum systems across various platforms. These developments encompass single-photon networks (*26*–*31*), cold atoms (*32*, *33*), trapped ions (*34*, *35*), superconducting circuits (*36*, *37*), and single nitrogen-vacancy centers (*38*, *39*). Furthermore, important research has demonstrated the evolution of quantum non-Hermitian systems in nuclear magnetic resonance quantum systems (*40*, *41*). Non-Hermitian operators have also been investigated as observables for quantum estimation (*42*). Using the non-Hermitian quantum systems for quantum parameter estimation, however, is still a largely unexplored area.

Extensive research has been devoted to achieving the coveted Heisenberg scaling in the realm of Hermitian systems (*43*–*46*). Various strategies have been explored, with the parallel scheme gaining prominence. This approach leverages entangled states as input to achieve Heisenberg precision (*47*–*49*). However, the preparation of high-quality, large entangled states poses a substantial challenge. As an alternative, the direct sequential scheme has emerged as another viable avenue to attain Heisenberg scaling without relying on entangled probe states (*50*–*53*). Substantial progress has been made in understanding the conditions necessary for achieving Heisenberg scaling in Hermitian systems under both the parallel and sequential schemes (*54*, *55*). For non-Hermitian systems in the quantum regime, such understanding is still very limited.

In this work, we investigate non-Hermitian quantum parameter estimation both theoretically and experimentally. First, we propose a concise expression for the QFI that is applicable to general non-Hermitian Hamiltonians. This expression allows us to analyze the estimation precision of non-Hermitian systems. We find that Heisenberg scaling, characterized by an inverse scaling with time *t*^−1^, can be achieved in non-Hermitian systems. Furthermore, we derive the condition for optimal measurements based on the proposed QFI expression. By identifying the optimal measurement strategy, we demonstrate that the estimation precision can reach the fundamental limit known as the quantum Cramér-Rao bound (QCRB). To experimentally validate our theoretical findings, we construct a non-unitary evolution governed by a PT-symmetric non-Hermitian Hamiltonian and estimate the associated parameters. By using the condition for optimal measurements obtained from our theoretical analysis, we achieve estimation precision that matches well with the QCRB. The experimental results reveal that the precision follows Heisenberg scaling *t*^−1^ for both multiplicative and non-multiplicative Hamiltonians. Our theory is universally applicable and independent of the symmetries of non-Hermitian Hamiltonians. This research not only enriches our understanding of non-Hermitian systems but also opens up exciting avenues for Heisenberg-limited quantum metrology.

## RESULTS

### QFI for general non-Hermitian Hamiltonians

The precision of a quantum system is theoretically limited by the QCRB, as given by ${\left(\Delta \hat{\theta }\right)}^{2}\geq 1/\left(nF_{\theta }\right)$ (*56*–*61*). Here, θ is the unknown parameter to be estimated, ${\left(\Delta \hat{\theta }\right)}^{2}$ is the variance of an unbiased estimator $\hat{\theta }$, *n* is the number of the measurements, and F_θ_ is QFI that characterizes the optimal estimation precision. For a multiplicative non-Hermitian Hamiltonian ${\hat{H}}_{0}=\hat{G}s$, where $\hat{G}$ is the generator and *s* is the parameter, the evolution of the system is described by the operator ${\hat{U}}_{\theta }=e^{-i{\hat{H}}_{0}t}=e^{-i\hat{G}\theta }$ (*40*, *62*), where θ = *st*. If the evolution time *t* is constant, then estimating θ is equivalent to estimating *s*.

In the case of a pure initial probe state, ∣ψ_0_〉〈ψ_0_∣, the QFI for estimating θ can be expressed as $F_{\theta }=4\left({\langle {\hat{G}}^{†}\hat{G}\rangle }_{\theta }-{\langle {\hat{G}}^{†}\rangle }_{\theta }{\langle \hat{G}\rangle }_{\theta }\right)$ (*63*), where ${\langle \hat{G}\rangle }_{\theta }=\langle \varphi_{\theta }|\hat{G}|\varphi_{\theta }\rangle$ represents the expectation value of the normalized output state, and $|\varphi_{\theta }\rangle ={\hat{U}}_{\theta }|\psi_{0}\rangle /\sqrt{\langle \psi_{0}|{\hat{U}}_{\theta }^{†}{\hat{U}}_{\theta }|\psi_{0}\rangle }$ represents the normalized output state (*64*). We refer the interested readers to the Supplementary Materials for the justification of such normalization.

For general non-multiplicative non-Hermitian Hamiltonians, the QFI cannot be expressed in the form mentioned earlier. In this work, we propose a general expression for the QFI that is applicable to both multiplicative and non-multiplicative non-Hermitian Hamiltonians. Consider a general non-Hermitian Hamiltonian ${\hat{H}}_{\alpha }$ that does not necessary takes the multiplicative form, the evolution operator is given by ${\hat{U}}_{\alpha }=e^{-i{\hat{H}}_{\alpha }t}$. The generator of the parameter α is denoted as ${\hat{h}}_{\alpha }=i\left(\partial_{\alpha }{\hat{U}}_{\alpha }\right){\hat{U}}_{\alpha }^{-1}$. On the basis of this, we can write the QFI as

$$F_{\alpha }=4\left({\langle {\hat{h}}^{†}\hat{h}\rangle }_{\alpha }-{\langle {\hat{h}}^{†}\rangle }_{\alpha }{\langle \hat{h}\rangle }_{\alpha }\right)$$ (1)

This expression provides a more general way to analyze the QFI for non-Hermitian Hamiltonians, regardless of whether they take the multiplicative form or not. One specific application of this expression is the analysis of enhanced or reduced sensitivity near EPs. By using this expression, we can deduce that the sensitivity of quantum sensors is reduced near EPs for two-level multiplicative non-Hermitian Hamiltonians. However, for non-multiplicative non-Hermitian Hamiltonians, the sensitivity may be enhanced and is affected by the modulus of the difference ∣Δλ∣ between two eigenvalues of $\hat{h}$ near the EP. This expression thus opens up avenues for the study of quantum metrology near EPs. Detailed derivations and discussions of Eq. 1 can be found in the Supplementary Materials. It should be noted that, when the Hamiltonian is Hermitian, this expression reduces to the previously reported form in (*58*–*61*) for Hermitian systems.

In addition to the previous analysis, with the expression for the QFI given in Eq. 1, we can further explore whether the Heisenberg scaling can be achieved in non-Hermitian systems using the direct sequential scheme. While the sequential scheme has been well-established as a means to attain Heisenberg scaling of 1/*t* in Hermitian systems, the applicability of this scaling to non-Hermitian systems has remained uncertain due to the previous formulation of the QFI using the expression F_α_ = 4(〈∂_α_φ_α_∣∂_α_φ_α_〉 − ∣〈∂_α_φ_α_∣φ_α_〉∣^2^). However, with the expression for the QFI provided in Eq. 1, we can now delve into the possibility of achieving the Heisenberg scaling in non-Hermitian systems through the direct sequential scheme. Specifically in the case of multiplicative non-Hermitian Hamiltonian where the evolution operator is given by $\hat{U}=e^{-i{\hat{H}}_{0}t}=e^{-i\hat{G}\mathrm{ts}}$ (estimating the parameter *s*), the generator of the parameter *s* is $\hat{G}t$. Using Eq. 1, we can calculate the QFI as $F_{s}=4t^{2}\left({\langle {\hat{G}}^{†}\hat{G}\rangle }_{s}-{\langle {\hat{G}}^{†}\rangle }_{s}{\langle \hat{G}\rangle }_{s}\right)$. It becomes evident that the precision of the estimation, given by $\sigma \left(\hat{s}\right)\geq 1/\sqrt{F_{s}}$, achieves the Heisenberg scaling of *t*^−1^.

Achieving the ultimate precision in quantum metrology, as quantified by the QCRB, requires the identification of optimal measurement strategies. It is well-known that one of the optimal measurements is the projections on the eigenbasis of the symmetric logarithmic derivative (SLD) operator *L*_α_ (*58*–*61*), which can be obtained as *L*_α_ = 2(∣*∂*_α_φ_α_〉〈φ_α_∣ + ∣φ_α_〉〈*∂*_α_φ_α_∣) for pure state. However, it is worth noting that the optimal measurement may not be unique, and the computation of the SLD may not be easy using the equation $\left({\hat{L}}_{\alpha }{\tilde{\rho }}_{\alpha }+{\tilde{\rho }}_{\alpha }{\hat{L}}_{\alpha }\right)/2=\partial_{\alpha }{\tilde{\rho }}_{\alpha }$, where ${\tilde{\rho }}_{\alpha }$ is the normalized output density matrix.

In the pursuit of optimal measurements in non-Hermitian systems, a condition has been proposed in (*63*) for multiplicative Hamiltonians. Here, we further generalize this condition to encompass general non-Hermitian Hamiltonians. Consider a Hermitian operator $\hat{A}$ as the observable with $\delta \hat{A}=\hat{A}-{\langle \hat{A}\rangle }_{\alpha }$ and ${\left(\Delta \hat{A}\right)}^{2}={\langle \delta {\hat{A}}^{†}\delta \hat{A}\rangle }_{\alpha }={\langle {\hat{A}}^{†}\hat{A}\rangle }_{\alpha }-{\langle {\hat{A}}^{†}\rangle }_{\alpha }{\langle \hat{A}\rangle }_{\alpha }$, the precision of the parameter estimation can be characterized via the error propagation formula ${\left(\Delta \alpha \right)}^{2}={\left(\Delta \hat{A}\right)}^{2}/\left(n{|\partial_{\alpha }{\langle \hat{A}\rangle }_{\alpha }|}^{2}\right)$ (*65*, *66*). Using the non-Hermitian uncertainty relationship ${\left(\Delta \hat{A}\right)}^{2}{\left(\Delta \hat{B}\right)}^{2}\geq |{\langle {\hat{A}}^{†}\hat{B}\rangle }_{\alpha }-{\langle {\hat{A}}^{†}\rangle }_{\alpha }{\langle \hat{B}\rangle }_{\alpha }|$ (*67*–*71*) by taking $\hat{B}$ as $\hat{h}$, we can obtain ${\left(\Delta \hat{A}\right)}^{2}{\left(\Delta \hat{h}\right)}^{2}\geq {|\partial_{\alpha }{\langle \hat{A}\rangle }_{\alpha }|}^{2}/4$ (see the Supplementary Materials). This inequality provides a lower bound on the variance of the estimator given by

$${\left(\Delta \alpha \right)}^{2}=\frac{{\left(\Delta \hat{A}\right)}^{2}}{n{|\partial_{\alpha }{\langle \hat{A}\rangle }_{\alpha }|}^{2}}\geq \frac{{\left(\Delta \hat{A}\right)}^{2}}{4n{\left(\Delta \hat{A}\right)}^{2}{\left(\Delta \hat{h}\right)}^{2}}=\frac{1}{nF_{\alpha }}$$ (2)

which is exactly the QCRB. This bound represents the fundamental limit on the precision of parameter estimation. The bound is saturated if and only if the observable $\hat{A}$ satisfies

∣f〉=ic∣g〉 (3)

where $|f\rangle =\delta \hat{h}|\varphi_{\alpha }\rangle$, $|g\rangle =\delta \hat{A}|\varphi_{\alpha }\rangle$, and *c* is a real number. This condition specifies the relationship between the observables $\hat{A}$ and $\hat{h}$ required to saturate the QCRB. By satisfying the condition in Eq. 3, one can attain optimal measurements in non-Hermitian systems, thereby achieving the ultimate precision allowed by the QCRB. This generalized condition opens up possibilities for designing optimal measurement strategies.

### Model of the experiment system

For the experiment, we consider a PT-symmetric non-Hermitian Hamiltonian given by

$${\hat{H}}_{\mathrm{PT}}=s\left(\begin{array}{cc} i\text{sin}\alpha & 1\\ 1 & -i\text{sin}\alpha \end{array}\right)$$ (4)

where *s* and α are real parameters. In this case, we assume that the PT symmetry is not broken, so we have 0 < α < π/2. The eigenvalues of ${\hat{H}}_{\mathrm{PT}}$ are λ_±_ = ± *s* cos α, and the corresponding normalized eigenstates are $|\lambda_{+}\rangle ={\left(e^{i\alpha /2},e^{-i\alpha /2}\right)}^{T}/\sqrt{2}$ and $|\lambda_{-}\rangle ={\left(e^{-i\alpha /2},-e^{i\alpha /2}\right)}^{T}/\sqrt{2}$.

**Fig. 1.**
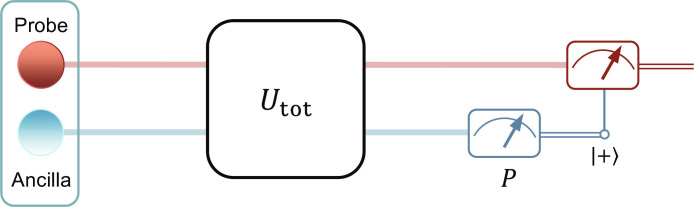
Post-selected scheme for the non-unitary evolution. The operator ${\hat{U}}_{\text{tot}}$ is an unitary evolution, and we effectively obtain the evolution $\hat{U}\prime_{\mathrm{PT}}={F\hat{U}}_{\mathrm{PT}}$ of the *PT*-symmetric Hamiltonian ${\hat{H}}_{\mathrm{PT}}$ for probe qubit after post-selection.

Furthermore, the non-unitary evolution operator is given by

$$\begin{array}{c} \begin{array}{cc} {\hat{U}}_{\mathrm{PT}} & =e^{-i{\hat{H}}_{\mathrm{PT}}t}\\ & =\frac{1}{\text{cos}\alpha }[\begin{array}{cc} \text{cos}(\mathrm{ts}\text{cos}\alpha -\alpha ) & -i\text{sin}(\mathrm{ts}\text{cos}\alpha )\\ -i\text{sin}(\mathrm{ts}\text{cos}\alpha ) & \text{cos}(\mathrm{ts}\text{cos}\alpha +\alpha ) \end{array}] \end{array} \end{array}$$ (5)

where *t* represents time. This non-unitary evolution can be accomplished using two-qubit dilated systems, which consist of a probe qubit and an ancilla qubit. The circuit representation of the total evolution ${\hat{U}}_{\text{tot}}$ for the two qubits is illustrated in Fig. 1. After post-selecting the ancilla qubit, the probe qubit undergoes a non-unitary evolution. However, it is essential to note that this non-unitary evolution occurs with a certain probability conditioned on the post-selection, which introduces a loss of a portion of states (*26*–*29*, *72*). Even when accounting for this loss, the Heisenberg scaling is still achievable.

The Heisenberg precision can be achieved for the estimation of *s* because the Hamiltonian ${\hat{H}}_{\mathrm{PT}}$ is in a multiplicative form with respect to *s*. When the probe state is initially in the state ∣ψ_0_〉 = ∣0〉, the QFI can be obtained as

$$F_{s}\left(t\right)=\frac{4t^{2}{\text{cos}}^{4}\alpha }{{\left[-1+\text{sin}\;\alpha \text{sin}\left(\alpha -2\mathrm{st}\text{cos}\alpha \right)\right]}^{2}}$$ (6)

This expression provides the QFI for the parameter *s* at a general time *t*, which achieves the Heisenberg scaling. For the estimation of α where the non-Hermitian Hamiltonians ${\hat{H}}_{\mathrm{PT}}$ does not take the multiplicative form, we can use Eq. 1 to obtain the QFI as

$$F_{\alpha }\left(t\right)={\left[\frac{1-\text{sec}\;\alpha \text{cos}\left(\alpha -2\mathrm{st}\;\text{cos}\;\alpha \right)+2\mathrm{st}\;\text{sin}\;\alpha }{\text{sec}\;\alpha -\text{sin}\left(\alpha -2\mathrm{st}\;\text{cos}\;\alpha \right)\text{tan}\;\alpha }\right]}^{2}$$ (7)

This also achieves the Heisenberg scaling *t*^2^. More detailed analysis and information can be found in the Supplementary Materials.

In addition, to illustrate the generality of Eq. 1, we investigate a broader scenario involving a non-Hermitian Hamiltonian ${\hat{H}}_{\kappa }=\kappa |0\rangle \langle 1|+|1\rangle \langle 0|$ without special symmetries. The experimental precision also achieves Heisenberg scaling, aligning with the theoretical analysis. The details are presented in the Supplementary Materials.

### Experimental setup and results

**Fig. 2.**
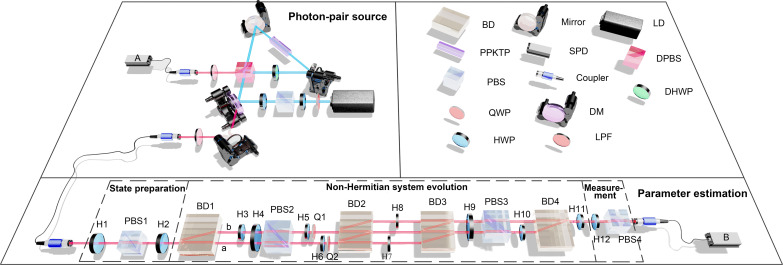
Schematic illustration of the experimental setup. Photons pair are generated by a periodically poled potassium titania phosphate (PPKTP) crystal, and the single target photon is heralded by trigger photon and prepared as probe state. The probe states are purified and rotated respectively by half-wave plate (HWP) and polarization beam splitter (PBS) in the module of state preparation and then evolve in the module non-Hermitian system evolution, and the parameter α is determined by H5 and H6. The output states after evolution are measured by PBS and HWP in the module of measurement.QWP, quarter-wave plate; SPD, single photon detector; DM, dichroic mirror; LPF, long pass filter; LD, laser diode; DPBS, dichroic polarization beam splitter; DHWP, dichroic half-wave plate.

The experimental setup, as depicted in Fig. 2, consists of four modules: (i) Photon pair source: A periodically poled potassium titanyl phosphate crystal is pumped by a 405-nm laser to generate photon pairs through the process of type II phase-matched spontaneous parametric down-conversion (*73*). One of the photons, called the target photon, serves as the qubit carrier for the non-Hermitian system evolution operation $\hat{U}\prime_{\mathrm{PT}}$. The other photon acts as a trigger signal, referred to as the trigger photon. To ensure data accuracy and reduce environmental interference, we record the coincidence count between photon counter A (trigger photon) and B (target photon), with a coincidence window of 1 ns. (ii) State preparation: Photons’ polarizations are used to encode states, where a horizontally polarized state ∣*H*〉 corresponds to ∣0〉, and a vertically polarized state ∣*V*〉 corresponds to ∣1〉. To prepare the target photon, it undergoes a rotation and purification process using half-wave plate 1 (H1) and polarization beam splitter 1 (PBS1), respectively. The target photon is initially prepared in the horizontal state ∣0〉 and can be further prepared as an arbitrary linear polarization pure probe state ∣ψ_0_〉 = cos 2ϕ∣0〉 + sin 2ϕ∣1〉 with the help of H2. (iii) Non-Hermitian system evolution: The non-Hermitian system evolution is achieved by using an ancilla qubit and the projection operation (for post-selection) (*26*). The detailed construction is described in Materials and Methods. In practical experiments, the non-unitary evolution is efficiently simulated in an open system by implementing a projection measurement on the ancilla qubit. The actual realized evolution operator is $\hat{U}\prime_{\mathrm{PT}}={F\hat{U}}_{\mathrm{PT}}$, where *F* is a function of the estimated parameter. Note that the evolution operator ${\hat{U}}_{\mathrm{PT}}$ multiplied by a function of the estimated parameter does not affect the expression of QFI, and the proof can be found in Materials and Methods. (iv) Measurement: This module consists of HWP and a PBS. Different eigenbases can be used to perform projective measurements. In this case, we choose the measurement $\hat{A}=|0\rangle \langle 0|$, which is the optimal measurement when the input probe state is ∣0〉. We also experimentally demonstrate the conditions for optimal measurements (see the Supplementary Materials for detail).

**Fig. 3.**
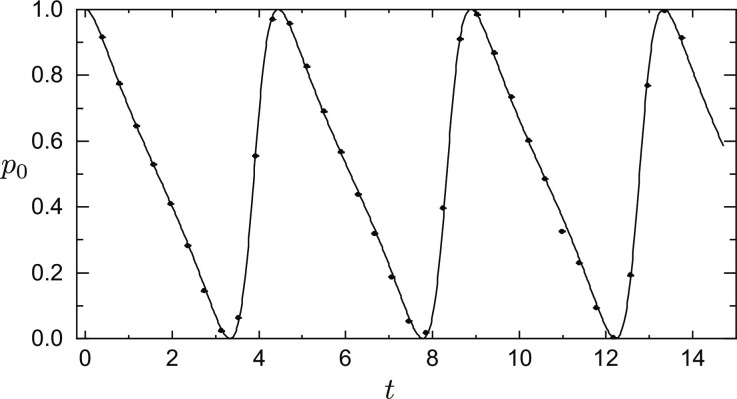
The probabilities of measurement outcomes for varying *t*. The black dots represent the experimentally measured data of *p*_0_ for varying *t*. In addition, we set *s* = 1, α = π/4, the measurement performed is $\hat{A}=|0\rangle \langle 0|$, and the probe state is ∣ψ_0_〉 = ∣0〉. The black solid line is the theoretical value of $p_{0}=\langle \varphi |\hat{A}|\varphi \rangle$, and the data points match well with the theoretical curve.

On the basis of the theoretical results that we discussed earlier, we conducted an experiment to achieve the precision with Heisenberg scaling. In this experiment, we prepared the initial state as ∣ψ_0_〉 = ∣0〉 and estimated the parameters *s* and α with the optimal measurement $\hat{A}$ for different values of *t*. The true values of the parameters are *s* = 1 and α = π/4. We performed *n* = 1500 to 2000 measurements to obtain the probabilities $p_{0}=\langle \varphi |\hat{A}|\varphi \rangle$ for each evolved probe state, where ∣φ〉 is the normalized final state. The experimental results, as shown in Fig. 3, are in agreement with the theoretical probabilities. This agreement validates, to a substantial extent, the accuracy of the evolution matrix $\hat{U}\prime_{\mathrm{PT}}$ used in the experiment.

**Fig. 4.**
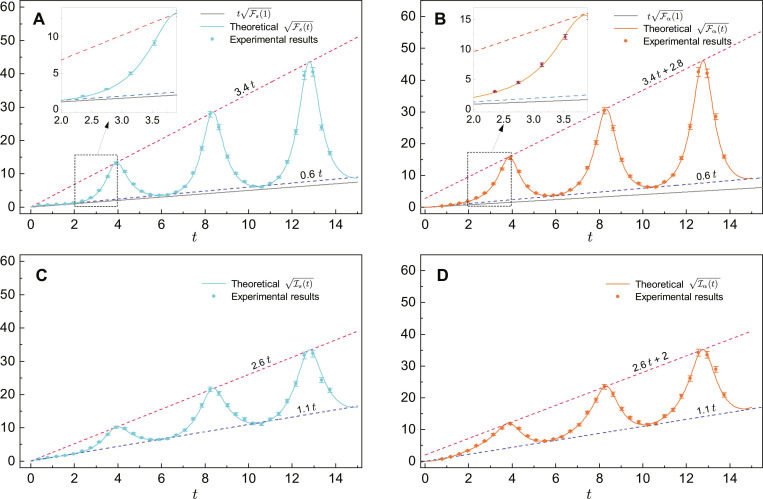
QFI for varying time *t*. The probe state is set as ∣0〉, the measurement performed is $\hat{A}$, and the condition for optimal measurements is satisfied. The practical values of *s* and α that we set are 1 and π/4. (**A**) The square root of QFI when *s* is estimated; the green dots are the experimental data, and the green solid line is the theoretical value of $\sqrt{F_{s}\left(t\right)}$. (**B**) The square root of QFI when α is estimated; the orange dots are the experimental data, and the orange solid line is the theoretical value of $\sqrt{F_{\alpha }\left(t\right)}$. (**C**) The QFI multiplied by normalized coefficient *K_s_*. (**D**) The QFI multiplied by normalized coefficient *K*_α_.

To obtain the statistical information of the estimation, we performed 1000 maximum likelihood estimates and obtained the distributions of the estimators $\hat{s}$ and $\hat{\alpha }$ separately. In Fig. 4 (A and B), we compare the experimental precision $1/\sigma \left(\hat{s}\right)$ and $1/\sigma \left(\hat{\alpha }\right)$ with the theoretical optimal estimation precision $\sqrt{F_{s}}$ and $\sqrt{F_{\alpha }}$, where $\sigma \left(\hat{s}\right)$ and $\sigma \left(\hat{\alpha }\right)$ are the SDs of the experimental estimation results. To compare with the theoretical results, we multiplied the coefficient $\sqrt{n}$ by the SD obtained in the experiment. It is important to note that the results shown in Fig. 4 (A and B) correspond to the precision of a single measurement. The experimental precision matches well with the theoretical estimation precision for both multiplicative and non-multiplicative Hamiltonians.

In non-Hermitian systems, the estimation precision of successful detection events is characterized by the QFI. However, to determine the ultimate precision for a given resource of probe states, it is necessary to multiply the QFI by the normalization coefficient *K* of the output state. This can be expressed as $\sqrt{I_{s}}=\sqrt{KF_{s}}$ and $\sqrt{I_{\alpha }}=\sqrt{KF_{\alpha }}$ (*63*). The normalization coefficient *K* is a periodic function, but it does not affect the overall estimation precision, which still achieves Heisenberg scaling. As shown in Fig. 4 (C and D), the growth of $\sqrt{I_{s}}$ and $\sqrt{I_{\alpha }}$ follows a scaling of *t*, with only a decrease in the oscillation amplitude.

**Fig. 5.**
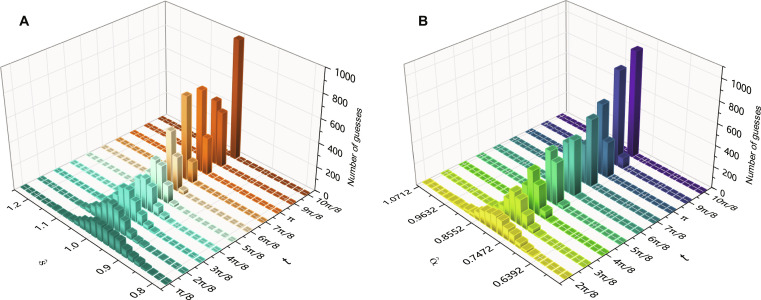
The distribution of estimator $\hat{s}$ and $\hat{\alpha }$ for varying *t*. The distribution becomes more centralized as time *t* increases (QFI is increased). (**A**) The distribution of $\hat{s}$. (**B**) The distribution of $\hat{\alpha }$.

The histograms of parameter estimation results for two estimators are also plotted, as shown in Fig. 5 (A and B). As indicated in Fig. 4, the estimation precision gradually improves within the range of *t* from *0* to 10π/8. Consequently, the distribution of the estimator becomes more centralized over time. In Fig. 5, it is observed that, when *t* is small, the center of the experimental distribution is noticeably larger than the theoretical value. This discrepancy arises due to the error in the constructed evolution, although the error itself is relatively small (as shown in Fig. 3). When the QFI is small, even a slight error in the probability of measurement outcomes can result in a notable error in the estimation of the parameter. Additional results can be found in the Supplementary Materials.

## DISCUSSIONS

One of the main goals in quantum metrology is to achieve the Heisenberg scaling, surpassing the classical limit. Recent studies on systems with Markovian noises have identified the conditions to achieve the Heisenberg scaling (*54*, *55*). These conditions show that the Heisenberg scaling is not attainable with generic Markovian noises (*74*). The demonstration of the Heisenberg scaling in non-Hermitian systems presented in this work opens up avenues for identifying systems capable of achieving this scaling. As we have shown, the QFI exhibits an oscillatory behavior as it increases with time in non-Hermitian systems. This phenomenon is paralleled by the periodic oscillation of state distinguishability in non-Hermitian systems (*28*, *75*). These oscillations result from the flow of information back from the environment, indicating non-Markovian behavior that exceeds the scope of previous research on achieving the Heisenberg scaling within Markovian dynamics (*54*, *55*). Although dealing with general non-Markovian systems can be challenging, the presence of non-Markovian behavior in non-Hermitian systems provides possibilities for the identification of systems that can achieve the Heisenberg scaling. By examining the relationship between these oscillatory behaviors and the attainment of the Heisenberg scaling, we anticipate gaining deeper insights into the interplay between quantum metrology and non-Hermitian physics.

In summary, we have introduced a formulation of QFI for general non-Hermitian Hamiltonians, enabling the distinction between systems with enhanced and reduced sensitivity near EPs. This provides a unique perspective for the study of quantum metrology in the vicinity of EPs. We have demonstrated that the Heisenberg scaling can be achieved both theoretically and experimentally in non-Hermitian systems. In addition, we have derived conditions for optimal measurements, which are applicable to both Hermitian and non-Hermitian systems. Building on this theoretical framework, we have implemented non-unitary evolutions governed by two non-Hermitian Hamiltonians and investigated parameter estimation for these evolutions. We have achieved the Heisenberg scaling for both parameter-independent and parameter-dependent Hamiltonians, with the estimation also reaching the QCRB. The experimental results closely match the theoretical model. Our theory does not make any specific assumptions about the Hamiltonian, and it remains valid for non-Hermitian Hamiltonians without special symmetries. This work represents a notable advancement in both theoretical and experimental research on quantum metrology in non-Hermitian systems.

## MATERIALS AND METHODS

### Implementation of the non-Hermitian system evolution $\hat{U}\prime_{\mathrm{PT}}$

The probe state is prepared as ∣ψ_0_〉 = cos 2ϕ∣0〉 + sin 2ϕ∣1〉; the photon is separated into two paths by beam displacer 1 (BD1), which introduces the ancilla qubit of path space (∣*a*〉 represents the path *a* and ∣*b*〉 represents the path *b*); the horizontal component remains unchanged (path *a*), while the vertical component is deflected into path *b*. The horizontal and vertical components are respectively prepared as $|\varphi_{H}\rangle =|\psi_{H}\rangle /\sqrt{\langle \psi_{H}|\psi_{H}\rangle }$ and $|\varphi_{V}\rangle =|\psi_{V}\rangle /\sqrt{\langle \psi_{V}|\psi_{V}\rangle }$ by H5, Q1, H6, and Q2, where $|\psi_{H}\rangle ={\hat{U}}_{\mathrm{PT}}|0\rangle$ and $|\psi_{V}\rangle ={\hat{U}}_{\mathrm{PT}}|1\rangle$. Last, ∣φ*_H_*〉 and ∣φ*_V_*〉 would be recombined into one path at the output port of the non-Hermitian system evolution, resulting in a loss of photons due to post-selection. As a result, the probe state becomes *F*(cos 2ϕ∣φ*_H_*〉 + sin 2ϕ∣φ*_V_*〉). However, the target output state is *F*(cos 2ϕ∣ψ*_H_*〉 + sin 2ϕ∣ψ*_V_*〉), and it should be noticed that the gain or loss of two components can be different, i.e., 〈ψ*_H_*∣ψ*_H_*〉 ≠ 〈ψ*_V_*∣ψ*_V_*〉, but 〈φ*_H_*∣φ*_H_*〉 = 〈φ*_V_*∣φ*_V_*〉 = 1. To realize it, we add a sub-module consisting of H3, H4, and PBS2, which could control components of ∣φ*_H_*〉 and ∣φ*_V_*〉 in two paths. Therefore, before BD2, the probe state is changed to

$$p\;\text{cos}2\phi |\varphi_{H}\rangle |a\rangle +q\text{sin}2\phi |\varphi_{V}\rangle |b\rangle$$ (8)

where *p* = sin 2(ϕ_1_ − ϕ_2_) and *q* = cos 2ϕ_2_ are controlled by H3 (ϕ_1_) and H4 (ϕ_2_), and *p*^2^/*q*^2^ = 〈ψ*_H_*∣ψ*_H_*〉/〈ψ*_V_*∣ψ*_V_*〉. The horizontal and vertical components of ∣φ*_H_*〉∣*a*〉 and ∣φ*_V_*〉∣*b*〉 are separated by BD2 and then recombined by H7, H8, and BD3. The post-selection is realized by performing projection operator $\hat{P}=\left(|a\rangle +|b\rangle \right)(\langle a|+\langle b|)/2$ on ancilla qubit, and the projection operator is constructed by PBS3 and H9 (22.5^∘^). After H10, BD4, and H11, two paths are combined into one path, the output state of probe qubit lastly can be written as

$$\begin{array}{c} \begin{array}{cc} \hat{U}\prime_{\mathrm{PT}}|\psi_{0}\rangle & =\frac{1}{\sqrt{2}}\left(p\text{cos}2\phi |\varphi_{H}\rangle +q\text{sin}2\phi |\varphi_{V}\rangle \right)\\ & =\frac{1}{\sqrt{2}}\left(\frac{p\text{cos}2\phi |\psi_{H}\rangle }{\sqrt{\langle \psi_{H}|\psi_{H}\rangle }}+\frac{q\text{sin}2\phi |\psi_{V}\rangle }{\sqrt{\langle \psi_{V}|\psi_{V}\rangle }}\right)\\ & =F\left(\text{cos}2\phi |\psi_{H}\rangle +\text{sin}2\phi |\psi_{V}\rangle \right) \end{array} \end{array}$$ (9)

where $F=p/\sqrt{\langle \psi_{H}|\psi_{H}\rangle }=q/\sqrt{\langle \psi_{V}|\psi_{V}\rangle }$. The theoretical output state is

$${\hat{U}}_{\mathrm{PT}}|\psi_{0}\rangle =\text{cos}2\phi |\psi_{H}\rangle +\text{sin}2\phi |\psi_{V}\rangle$$ (10)

Therefore, the actual evolution that we constructed is $\hat{U}\prime_{\mathrm{PT}}={F\hat{U}}_{\mathrm{PT}}$.

### Proof of the invariance of QFI

We can prove that multiplying the evolution operator with a scalar function, denoted as *F*(α), does not change the QFI of the normalized state. Let us consider the original expression of QFI with F_α_ = 4(〈*∂*_α_φ_α_∣*∂*_α_φ_α_〉 − ∣〈*∂*_α_φ_α_∣φ_α_〉∣^2^), where α is the parameter to be estimated. We can decompose the scalar function *F*(α) into its modulus and phase, *F*(α) = *R*(α)*e*^*if*(α)^. When the evolution operator is multiplied by *F*(α), it becomes $\hat{U}\prime \left(\alpha \right)=F\left(\alpha \right)\hat{U}\left(\alpha \right)=R\left(\alpha \right)e^{\text{if}\left(\alpha \right)}\hat{U}\left(\alpha \right)$. The normalized final state after the multiplication is given by

$$\begin{array}{c} \begin{array}{cc} |{\varphi \prime }_{\alpha }\rangle & =\frac{\hat{U}\prime \left(\alpha \right)|\psi_{0}\rangle }{\sqrt{\langle \psi_{0}|\hat{U}\prime {\left(\alpha \right)}^{†}\hat{U}\prime \left(\alpha \right)|\psi_{0}\rangle }}\\ & =\frac{e^{\text{if}\left(\alpha \right)}\hat{U}\left(\alpha \right)|\psi_{0}\rangle }{\sqrt{\langle \psi_{0}|\hat{U}{\left(\alpha \right)}^{†}\hat{U}\left(\alpha \right)|\psi_{0}\rangle }}=e^{\text{if}\left(\alpha \right)}|\varphi_{\alpha }\rangle \end{array} \end{array}$$ (11)

It can be observed that, if *F*(α) is a real function, then the normalized final state remains unchanged, and, consequently, the QFI does not change. However, if *F*(α) is a complex function, then there will be a phase difference *e*^*if*(α)^ between ∣φ′_α_〉 and ∣φ_α_〉, and this phase is also a function of α.

To simplify the explanation, let us consider this problem from a geometric standpoint. The QFI can also be defined in terms of the quantum geometric tensor (QGT). The QGT, which depends on a set of parameters denoted as *x* = (*x*_1_, *x*_2_, …) ∈ ℳ, represents a manifold of the quantum system. The QGT is defined as *Q*_μν_(*x*) = 〈∂_μ_φ(*x*)∣∂_ν_φ(*x*)〉 − 〈∂_μ_φ(*x*)∣φ(*x*)〉〈φ(*x*)∣∂_ν_φ(*x*)〉 (*76*–*78*), where ∂_μ_ = ∂/∂x_μ_, and we have a gauge-invariant metric given by *g*_μν_ = Re [*Q*_μν_]. This metric, *g*_μν_, remains invariant under gauge transformations of the form ∣φ′(*x*)〉 = *e*^*if*(*x*)^∣φ(*x*)〉. Therefore, the single parameter QFI is exactly the same as the gauge-invariant metric of a one-dimensional manifold α ∈ ℳ.

According to the gauge invariance, we know that *g*_μν_ is invariant under the gauge transformation ∣φ′(α)〉 = *e*^*if*(α)^∣φ(α)〉. Because F_α_ = 4Re[*Q*_αα_] = 4*g*_αα_, the QFI is also invariant when the evolution operator is multiplied by a function of α.

### Analysis of experimental imperfections and details

In our experimental setup, as depicted in Fig. 2, the optical path difference between BD1 and BD3 is very small, and H9 is set at an angle of 22.5^∘^, resulting in a Mach-Zehnder interference. This interference leads to the increase or decrease in the number of photons after post-selection when we input a superposition state of ∣0〉 and ∣1〉. Consequently, the accuracy of the non-Hermitian evolution is compromised. The fluctuation in the double-coincidence event rate during long-term experiments also affects the accuracy of the evolution. To minimize the interference, it is crucial to maintain a stable experimental environment.

In our experiment, the experimental double-coincidence event rate is approximately 15 kHz after the non-Hermitian system evolution. To obtain the probabilities of measurement outcomes, we measured the final states using both ∣0〉〈0∣ and ∣1〉〈1∣. We denote the coincidence events of ∣0〉〈0∣ and ∣1〉〈1∣ as *N*_0_ and *N*_1_, respectively. The probability of jumping into ∣0〉 is calculated as *p*_0_ = *N*_0_/(*N*_0_ + *N*_1_).

To mitigate the experimental errors caused by the variation in the double-coincidence event rate during long-term experiments, we recorded the coincidence events within a time window of 0.3 s. In addition, we changed the measurement every 500 data points. This approach reduces the fluctuations in the number of measurements between the two different projective operators.

### Error analysis

In our experiment, a estimation of the parameter α is based on *n* = 1500 to 2000 measurement outcomes. By repeating these *n* measurements *K* = 1000 times, we obtain 1000 estimation of α. On the basis of this set of estimation results, we could obtain the SD of the estimation $\sigma \left(\hat{\alpha }\right)$. The error of $\sigma \left(\hat{\alpha }\right)$, denoted as $\Delta \left[\sigma \left(\hat{\alpha }\right)\right]$ can be approximated by $\Delta \left[\sigma \left(\hat{\alpha }\right)\right]=\sigma \left(\hat{\alpha }\right)/\sqrt{2\left(K-1\right)}$ (*53*, *79*). According to QCRB, the experimental QFI depends on the $\sigma \left(\hat{\alpha }\right)$, so we have $\sqrt{F_{\alpha }}=1/\left[\sigma \left(\hat{\alpha }\right)\sqrt{n}\right]$. In addition, the error of $\sqrt{F_{\alpha }}$ is approximated by $\Delta \left(\sqrt{F_{\alpha }}\right)=\sqrt{F_{\alpha }}/\sqrt{2\left(K-1\right)}$, which is used to draw the error bar in Fig. 4. The error analysis corresponding to parameter *s* is the same.

## Supplementary Materials

### This PDF file includes:

Supplementary Text Figs. S1 to S10 Tables S1 to S7 References

## References

[1] C. M. Bender, S. Boettcher, Real spectra in non-hermitian Hamiltonians having 𝒫𝒯 symmetry. Phys. Rev. Lett. 80, 5243 (1998).

[2] C. M. Bender, D. C. Brody, H. F. Jones, Complex extension of quantum mechanics. Phys. Rev. Lett. 89, 270401 (2004). 10.1103/PhysRevLett.89.270401 12513185

[3] L. Feng, Z. J. Wong, R.-M. Ma, Y. Wang, X. Zhang, Single-mode laser by parity-time symmetry breaking. Science 346, 972–975 (2014). 25414307 10.1126/science.1258479

[4] H. Hodaei, M.-A. Miri, M. Heinrich, D. N. Christodoulides, M. Khajavikhan, Parity-time-symmetric microring lasers. Science 346, 975–978 (2014). 25414308 10.1126/science.1258480

[5] P. Miao, Z. Zhang, J. Sun, W. Walasik, S. Longhi, N. M. Litchinitser, L. Feng, Orbital angular momentum microlaser. Science 353, 464–467 (2016). 27471299 10.1126/science.aaf8533

[6] S. Longhi, 𝒫𝒯-symmetric laser absorber. Phys. Rev. A 82, 031801 (2010).

[7] Y. D. Chong, L. Ge, A. D. Stone, 𝒫𝒯-symmetry breaking and laser-absorber modes in optical scattering systems. Phys. Rev. Lett. 106, 093902 (2011). 21405622 10.1103/PhysRevLett.106.093902

[8] Y. Sun, W. Tan, H.-q. Li, J. Li, H. Chen, Experimental demonstration of a coherent perfect absorber with PT phase transition. Phys. Rev. Lett. 112, 143903 (2014). 24765965 10.1103/PhysRevLett.112.143903

[9] J. Doppler, A. A. Mailybaev, J. Böhm, U. Kuhl, A. Girschik, F. Libisch, T. J. Milburn, P. Rabl, N. Moiseyev, S. Rotter, Dynamically encircling an exceptional point for asymmetric mode switching. Nature 537, 76–79 (2016). 27454554 10.1038/nature18605

[10] H. Xu, D. Mason, L. Jiang, J. G. E. Harris, Topological energy transfer in an optomechanical system with exceptional points. Nature 537, 80–83 (2016). 27454555 10.1038/nature18604

[11] M. Kang, F. Liu, J. Li, Effective spontaneous 𝒫𝒯-symmetry breaking in hybridized metamaterials. Phys. Rev. A 87, 053824 (2013).

[12] M. Kang, J. Chen, Y. D. Chong, Chiral exceptional points in metasurfaces. Phys. Rev. A 94, 033834 (2016).

[13] S. Xiao, J. Gear, S. Rotter, J. Li, Effective PT-symmetric metasurfaces for subwavelength amplified sensing. New J. Phys. 18, 085004 (2016).

[14] R. Fleury, D. L. Sounas, A. Alù, Negative refraction and planar focusing based on parity-time symmetric metasurfaces. Phys. Rev. Lett. 113, 023903 (2014). 25062184 10.1103/PhysRevLett.113.023903

[15] B. Peng, Ş. K. Özdemir, F. Lei, F. Monifi, M. Gianfreda, G. L. Long, S. Fan, F. Nori, C. M. Bender, L. Yang, Parity-time-symmetric whispering-gallery microcavities. Nat. Phys. 10, 394–398 (2014).

[16] L. Feng, M. Ayache, J. Huang, Y.-L. Xu, M.-H. Lu, Y.-F. Chen, Y. Fainman, A. Scherer, Nonreciprocal light propagation in a silicon photonic circuit. Science 333, 729–733 (2011). 21817046 10.1126/science.1206038

[17] J. Wiersig, Enhancing the sensitivity of frequency and energy splitting detection by using exceptional points: Application to microcavity sensors for single-particle detection. Phys. Rev. Lett. 112, 203901 (2014).

[18] J. Wiersig, Sensors operating at exceptional points: General theory. Phys. Rev. A 93, 033809 (2016).

[19] Z.-P. Liu, J. Zhang, Ş. K. Özdemir, B. Peng, H. Jing, X.-Y. Lü, C.-W. Li, L. Yang, F. Nori, Y.-X. Liu, Metrology with 𝒫𝒯-symmetric cavities: Enhanced sensitivity near the 𝒫𝒯-phase transition. Phys. Rev. Lett. 117, 110802 (2016). 27661674 10.1103/PhysRevLett.117.110802

[20] W. Chen, Ş. K. Özdemir, G. Zhao, J. Wiersig, L. Yang, Exceptional points enhance sensing in an optical microcavity. Nature 548, 192–196 (2017). 28796206 10.1038/nature23281

[21] H. Hodaei, A. U. Hassan, S. Wittek, H. Garcia-Gracia, R. El-Ganainy, D. N. Christodoulides, M. Khajavikhan, Enhanced sensitivity at higher-order exceptional points. Nature 548, 187–191 (2017). 28796201 10.1038/nature23280

[22] H.-K. Lau, A. A. Clerk, Fundamental limits and non-reciprocal approaches in non-Hermitian quantum sensing. Nat. Commun. 9, 4320 (2018). 30333486 10.1038/s41467-018-06477-7 PMC6193019

[23] M. Zhang, W. Sweeney, C. W. Hsu, L. Yang, A. D. Stone, L. Jiang, Quantum noise theory of exceptional point amplifying sensors. Phys. Rev. Lett. 123, 180501 (2019). 31763922 10.1103/PhysRevLett.123.180501

[24] C. Chen, L. Jin, R.-B. Liu, Sensitivity of parameter estimation near the exceptional point of a non-Hermitian system. New J. Phys. 21, 083002 (2019).

[25] J. Wang, D. Mukhopadhyay, G. S. Agarwal, Quantum Fisher information perspective on sensing in anti-PT symmetric systems. Phys. Rev. Res. 4, 013131 (2022).

[26] J.-S. Tang, Y.-T. Wang, S. Yu, D.-Y. He, J.-S. Xu, B.-H. Liu, G. Chen, Y.-N. Sun, K. Sun, Y.-J. Han, C.-F. Li, G.-C. Guo, Experimental investigation of the no-signalling principle in parity-time symmetric theory using an open quantum system. Nat. Photonics 10, 642–646 (2016).

[27] Q. Li, C.-J. Zhang, Z.-D. Cheng, W.-Z. Liu, J.-F. Wang, F.-F. Yan, Z.-H. Lin, Y. Xiao, K. Sun, Y.-T. Wang, J.-S. Tang, J.-S. Xu, C.-F. Li, G.-C. Guo, Experimental simulation of anti-parity-time symmetric Lorentz dynamics. Optica 6, 67–71 (2019).

[28] Y.-T. Wang, Z.-P. Li, S. Yu, Z.-J. Ke, W. Liu, Y. Meng, Y.-Z. Yang, J.-S. Tang, C.-F. Li, G.-C. Guo, Experimental investigation of state distinguishability in parity-time symmetric quantum dynamics. Phys. Rev. Lett. 124, 230402 (2020). 32603176 10.1103/PhysRevLett.124.230402

[29] S. Yu, Y. Meng, J.-S. Tang, X.-Y. Xu, Y.-T. Wang, P. Yin, Z.-J. Ke, W. Liu, Z.-P. Li, Y.-Z. Yang, G. Chen, Y.-J. Han, C.-F. Li, G.-C. Guo, Experimental investigation of quantum 𝒫𝒯-enhanced sensor. Phys. Rev. Lett. 125, 240506 (2020). 33412046 10.1103/PhysRevLett.125.240506

[30] L. Xiao, X. Zhan, Z. H. Bian, K. K. Wang, X. Zhang, X. P. Wang, J. Li, K. Mochizuki, D. Kim, N. Kawakami, W. Yi, H. Obuse, B. C. Sanders, P. Xue, Observation of topological edge states in parity-time-symmetric quantum walks. Nat. Phys. 13, 1117–1123 (2017).

[31] L. Xiao, K. Wang, X. Zhan, Z. Bian, K. Kawabata, M. Ueda, W. Yi, P. Xue, Observation of critical phenomena in parity-time-symmetric quantum dynamics. Phys. Rev. Lett. 123, 230401 (2019). 31868428 10.1103/PhysRevLett.123.230401

[32] J. Li, A. K. Harter, J. Liu, L. de Melo, Y. N. Joglekar, L. Luo, Observation of parity-time symmetry breaking transitions in a dissipative Floquet system of ultracold atoms. Nat. Commun. 10, 855 (2019). 30787299 10.1038/s41467-019-08596-1 PMC6382795

[33] Y. Jiang, Y. Mei, Y. Zuo, Y. Zhai, J. Li, J. Wen, S. Du, Anti-parity-time symmetric optical four-wave mixing in cold atoms. Phys. Rev. Lett. 123, 193604 (2019). 31765185 10.1103/PhysRevLett.123.193604

[34] L. Ding, K. Shi, Q. Zhang, D. Shen, X. Zhang, W. Zhang, Experimental determination of 𝒫𝒯-symmetric exceptional points in a single trapped ion. Phys. Rev. Lett. 126, 083604 (2021). 33709727 10.1103/PhysRevLett.126.083604

[35] W.-C. Wang, Y.-L. Zhou, H.-L. Zhang, J. Zhang, M.-C. Zhang, Y. Xie, C.-W. Wu, T. Chen, B.-Q. Ou, W. Wu, H. Jing, P.-X. Chen, Observation of 𝒫𝒯-symmetric quantum coherence in a single-ion system. Phys. Rev. A 103, L020201 (2021).

[36] M. Naghiloo, M. Abbasi, Y. N. Joglekar, K. W. Murch, Quantum state tomography across the exceptional point in a single dissipative qubit. Nat. Phys. 15, 1232–1236 (2019).

[37] M. Partanen, J. Goetz, K. Y. Tan, K. Kohvakka, V. Sevriuk, R. E. Lake, R. Kokkoniemi, J. Ikonen, D. Hazra, A. Mäkinen, E. Hyyppä, L. Grönberg, V. Vesterinen, M. Silveri, M. Möttönen, Exceptional points in tunable superconducting resonators. Phys. Rev. B 100, 134505 (2019).

[38] Y. Wu, W. Liu, J. Geng, X. Song, X. Ye, C.-K. Duan, X. Rong, J. Du, Observation of parity-time symmetry breaking in a single-spin system. Science 364, 878–880 (2019). 31147518 10.1126/science.aaw8205

[39] W. Liu, Y. Wu, C.-K. Duan, X. Rong, J. Du, Dynamically encircling an exceptional point in a real quantum system. Phys. Rev. Lett. 126, 170506 (2021). 33988415 10.1103/PhysRevLett.126.170506

[40] C. M. Bender, D. C. Brody, H. F. Jones, B. K. Meister, Faster than Hermitian quantum mechanics. Phys. Rev. Lett. 98, 040403 (2007). 17358747 10.1103/PhysRevLett.98.040403

[41] C. Zheng, L. Hao, G. L. Long, Observation of a fast evolution in a parity-time-symmetric system. Phil. Trans. A Math. Phys. Eng. Sci. 371, 20120053 (2013). 10.1098/rsta.2012.0053 23509381

[42] J. Li, H. Liu, Z. Wang, X. X. Yi, Enhanced parameter estimation by measurement of non-Hermitian operators. AAPPS Bull. 33, 22 (2023).

[43] J. J. Bollinger, W. M. Itano, D. J. Wineland, D. J. Heinzen, Optimal frequency measurements with maximally correlated states. Phys. Rev. A 54, R4649–R4652 (1996). 9914139 10.1103/physreva.54.r4649

[44] V. Giovannetti, S. Lloyd, L. Maccone, Quantum-enhanced measurements: Beating the standard quantum limit. Science 306, 1330–1336 (2004). 15550661 10.1126/science.1104149

[45] V. Giovannetti, S. Lloyd, L. Maccone, Quantum metrology. Phys. Rev. Lett. 96, 010401 (2006). 16486424 10.1103/PhysRevLett.96.010401

[46] Q. Liu, Z. Hu, H. Yuan, Y. Yang, Optimal strategies of quantum metrology with a strict hierarchy. Phys. Rev. Lett. 130, 070803 (2023). 36867832 10.1103/PhysRevLett.130.070803

[47] T. Nagata, R. Okamoto, J. L. O’Brien, K. Sasaki, S. Takeuchi, Beating the standard quantum limit with four-entangled photons. Science 316, 726–729 (2007). 17478715 10.1126/science.1138007

[48] G. Y. Xiang, B. L. Higgins, D. W. Berry, H. M. Wiseman, G. J. Pryde, Entanglement-enhanced measurement of a completely unknown optical phase. Nat. Photonics 5, 43–47 (2011).

[49] R. Okamoto, H. F. Hofmann, T. Nagata, J. L. O’Brien, K. Sasaki, S. Takeuchi, Beating the standard quantum limit: Phase super-sensitivity of *N*-photon interferometers. New J. Phys. 10, 073033 (2008).

[50] B. L. Higgins, D. W. Berry, S. D. Bartlett, H. M. Wiseman, G. J. Pryde, Entanglement-free Heisenberg-limited phase estimation. Nature 450, 393–396 (2007). 18004379 10.1038/nature06257

[51] H. Yuan, C.-H. F. Fung, Optimal feedback scheme and universal time scaling for Hamiltonian parameter estimation. Phys. Rev. Lett. 115, 110401 (2015). 26406810 10.1103/PhysRevLett.115.110401

[52] D. Braun, G. Adesso, F. Benatti, R. Floreanini, U. Marzolino, M. W. Mitchell, S. Pirandola, Quantum-enhanced measurements without entanglement. Rev. Mod. Phys. 90, 035006 (2018).

[53] Z. Hou, R.-J. Wang, J.-F. Tang, H. Yuan, G.-Y. Xiang, C.-F. Li, G.-C. Guo, Control-enhanced sequential scheme for general quantum parameter estimation at the Heisenberg limit. Phys. Rev. Lett. 123, 040501 (2019). 31491234 10.1103/PhysRevLett.123.040501

[54] S. Zhou, M. Zhang, J. Preskill, L. Jiang, Achieving the Heisenberg limit in quantum metrology using quantum error correction. Nat. Commun. 9, 78 (2018). 29311599 10.1038/s41467-017-02510-3 PMC5758555

[55] R. Demkowicz-Dobrzański, J. Czajkowski, P. Sekatski, Adaptive quantum metrology under general Markovian noise. Phys. Rev. X 7, 041009 (2017).

[56] C. W. Helstrom, *Quantum Detection and Estimation Theory* (Academic Press, 1976).

[57] A. S. Holevo, *Probabilistic and Statistical Aspects of Quantum Theory* (North-Holland, 1982).

[58] G. Tóth, I. Apellaniz, Quantum metrology from a quantum information science perspective. J. Phys. A Math. Theor. 47, 42400 (2014).

[59] S. L. Braunstein, C. M. Caves, Statistical distance and the geometry of quantum states. Phys. Rev. Lett. 72, 3439–3443 (1994). 10056200 10.1103/PhysRevLett.72.3439

[60] S. L. Braunstein, C. M. Caves, G. J. Milburn, Generalized uncertainty relations: Theory, examples, and Lorentz invariance. Ann. Phys. 247, 135–173 (1996).

[61] S. Pang, T. A. Brun, Quantum metrology for a general Hamiltonian parameter. Phys. Rev. A 90, 022117 (2014).

[62] U. Günther, B. F. Samsonov, Naimark-dilated 𝒫𝒯-symmetric brachistochrone. Phys. Rev. Lett. 101, 230404 (2008). 19113530 10.1103/PhysRevLett.101.230404

[63] X. Yu, C. Zhang, Quantum parameter estimation of non-Hermitian systems with optimal measurements. Phys. Rev. A 108, 022215 (2023).

[64] D. C. Brody, E.-M. Graefe, Mixed-state evolution in the presence of gain and loss. Phys. Rev. Lett. 109, 230405 (2012). 23368172 10.1103/PhysRevLett.109.230405

[65] B. Yurke, S. L. McCall, J. R. Klauder, SU(2) and SU(1,1) interferometers. Phys. Rev. A 33, 4033–4054 (1986). 10.1103/physreva.33.4033 9897145

[66] S. F. Huelga, C. Macchiavello, T. Pellizzari, A. K. Ekert, M. B. Plenio, J. I. Cirac, Improvement of frequency standards with quantum entanglement. Phys. Rev. Lett. 79, 3865–3868 (1997).

[67] A. K. Pati, U. Singh, U. Sinha, Measuring non-Hermitian operators via weak values. Phys. Rev. A 92, 052120 (2015).

[68] M. J. W. Hall, A. K. Pati, J. Wu, Products of weak values: Uncertainty relations, complementarity, and incompatibility. Phys. Rev. A 93, 052118 (2016).

[69] D. Mondal, S. Bagchi, A. K. Pati, Tighter uncertainty and reverse uncertainty relations. Phys. Rev. A 95, 052117 (2017).

[70] B. Yu, N. Jing, X. Li-Jost, Strong unitary uncertainty relations. Phys. Rev. A 100, 022116 (2019).

[71] X. Zhao, C. Zhang, Uncertainty relations of non-hermitian operators: Theory and experimental scheme. Front. Phys. 10, 862868 (2022).

[72] G.-L. Long, General quantum interference principle and duality computer. Commun. Theor. Phys. 45, 825 (2006).

[73] T. Kim, M. Fiorentino, F. N. C. Wong, Phase-stable source of polarization-entangled photons using a polarization Sagnac interferometer. Phys. Rev. A 73, 012316 (2006).

[74] R. Demkowicz-Dobrzański, J. Kołodyński, M. Guçă, The elusive Heisenberg limit in quantum-enhanced metrology. Nat. Commun. 3, 1063 (2012). 22990859 10.1038/ncomms2067 PMC3658100

[75] K. Kawabata, Y. Ashida, M. Ueda, Information retrieval and criticality in parity-time-symmetric systems. Phys. Rev. Lett. 119, 19040 (2017). 10.1103/PhysRevLett.119.190401 29219512

[76] R. Cheng, Quantum geometric tensor (Fubini-Study metric) in simple quantum system: A pedagogical introduction. arXiv:1012.1337 (2013).

[77] C. Li, M. Chen, P. Cappellaro, A geometric perspective: Experimental evaluation of the quantum Cramer-Rao bound. arXiv:2204.13777 (2022).

[78] D. Brody, E.-M. Graefe, Information geometry of complex Hamiltonians and exceptional points. Entropy 15, 3361–3378 (2013).

[79] S. Ahn, J. A. Fessler, “Standard errors of mean, variance, and standard deviation estimators” (Technical Report, EECS Department, The University of Michigan, 2003), pp. 1–2.

[80] R. M. Wilcox, Exponential operators and parameter differentiation in quantum physics. J. Math. Phys. 8, 962–982 (1967).

