# Supplementary material for: Toward Heisenberg scaling in non-Hermitian metrology at the quantum regime
Source: Sci Adv. 2024 May 10;10(19):eadk7616. doi: 10.1126/sciadv.adk7616 (PMC11086624; doi:10.1126/sciadv.adk7616)
Supplement: Supplementary file 1 — Supplementary Text Figs. S1 to S10 Tables S1 to S7 References [file sciadv.adk7616_sm.pdf]

Science Advances
AAAS

Supplementary Materials for

Toward Heisenberg scaling in non-Hermitian metrology at the quantum regime

Xinglei Yu *et al.*

Corresponding author: Haidong Yuan, hdyuan@mae.cuhk.edu.hk; Chengjie Zhang, cjzhang@ustc.edu

Sci. Adv. **10**, eadk7616 (2024)
DOI: 10.1126/sciadv.adk7616

This PDF file includes:

Supplementary Text
Figs. S1 to S10
Tables S1 to S7
References

I. THEORY

A. Detailed derivation of QFI for general non-Hermitian Hamiltonians

Consider a non-Hermitian Hamiltonian $\hat{H}_\alpha$ with unknown parameter α, the evolution operator can be written as $\hat{U}_\alpha = e^{-i\hat{H}_\alpha t}$ (*40*), and the probe state is prepared to be $\rho_0 = |\psi_0\rangle\langle\psi_0|$. After evolution, the probe state becomes $\rho_\alpha = \hat{U}_\alpha \rho_0 \hat{U}_\alpha^\dagger = |\psi_\alpha\rangle\langle\psi_\alpha|$. When α is changed to $\alpha + d\alpha$, the evolution operator becomes $\hat{U}_{\alpha+d\alpha} \approx \hat{U}_\alpha + \partial_\alpha \hat{U}_\alpha d\alpha$, then we can further deduce the corresponding output state (*61*),

$$\begin{aligned}
\rho_{\alpha+d\alpha} &\approx (\hat{U}_\alpha + \partial_\alpha \hat{U}_\alpha d\alpha)\rho_0(\hat{U}_\alpha^\dagger + \partial_\alpha \hat{U}_\alpha^\dagger d\alpha) \\
&= [I + d\alpha(\partial_\alpha \hat{U}_\alpha)\hat{U}_\alpha^{-1}]\hat{U}_\alpha \rho_0 \hat{U}_\alpha^\dagger [I + d\alpha(\hat{U}_\alpha^\dagger)^{-1}(\partial_\alpha \hat{U}_\alpha^\dagger)] \\
&= (I - ihd\alpha)\rho_\alpha(I + i\hat{h}^\dagger d\alpha) \\
&\approx e^{-i\hat{h}d\alpha}\rho_\alpha e^{i\hat{h}^\dagger d\alpha},
\end{aligned} \tag{S1}$$

where

$$\hat{h} = i(\partial_\alpha \hat{U}_\alpha)\hat{U}_\alpha^{-1} \tag{S2}$$

is the local generator of the parametric translation of $\hat{U}_\alpha$ with respect to α.

In non-Hermitian systems, the evolution operator U_α is non-unitary, so the output state will no longer be normalized after evolution. However, as for measurement process, the probabilities of measurement outcomes sum up to 1. Therefore, the output state can be written as (*64*)

$$\tilde{\rho}_\alpha = |\varphi_\alpha\rangle\langle\varphi_\alpha| = \frac{\rho_\alpha}{\mathrm{Tr}\{\rho_\alpha\}} = \frac{\hat{U}_\alpha|\psi_0\rangle\langle\psi_0|\hat{U}_\alpha^\dagger}{\mathrm{Tr}\{\rho_\alpha\}}. \tag{S3}$$

the corresponding QFI is $\mathcal{F}_\alpha = 4(\langle\partial_\alpha\varphi_\alpha|\partial_\alpha\varphi_\alpha\rangle - |\langle\partial_\alpha\varphi_\alpha|\varphi_\alpha\rangle|^2)$. Then, we rewrite the QFI and associate it with h_α. Let $K_\alpha = \mathrm{Tr}\{\rho_\alpha\}$, then we have $|\varphi_\alpha\rangle = \hat{U}_\alpha|\psi_0\rangle/\sqrt{K_\alpha}$, the first term

$\langle\partial_\alpha\varphi_\alpha|\partial_\alpha\varphi_\alpha\rangle$ can be expand as follow

$$\begin{aligned}\langle\partial_\alpha\varphi_\alpha|\partial_\alpha\varphi_\alpha\rangle &= \partial_\alpha(\frac{\langle\psi_0|\hat{U}_\alpha^\dagger}{\sqrt{K_\alpha}})\partial_\alpha(\frac{\hat{U}_\alpha|\psi_0\rangle}{\sqrt{K_\alpha}}) \\ &= \langle\psi_0|\frac{(\partial_\alpha\hat{U}_\alpha^\dagger)\sqrt{K_\alpha}-\frac{(\partial_\alpha K_\alpha)}{2\sqrt{K_\alpha}}\hat{U}_\alpha^\dagger}{K_\alpha}\cdot\frac{\sqrt{K_\alpha}(\partial_\alpha\hat{U}_\alpha)-\frac{(\partial_\alpha K_\alpha)}{2\sqrt{K_\alpha}}\hat{U}_\alpha}{K_\alpha}|\psi_0\rangle \\ &= \frac{\langle\psi_0|(\partial_\alpha\hat{U}_\alpha^\dagger)(\partial_\alpha\hat{U}_\alpha)|\psi_0\rangle}{K_\alpha}-\frac{\partial_\alpha K_\alpha}{2K_\alpha^2}\langle\psi_0|[\hat{U}_\alpha^\dagger(\partial_\alpha\hat{U}_\alpha)+(\partial_\alpha\hat{U}_\alpha^\dagger)\hat{U}_\alpha]|\psi_0\rangle+\frac{(\partial_\alpha K_\alpha)^2}{4K_\alpha^2}.\end{aligned} \tag{S4}$$

Similarly, the second term $|\langle\partial_\alpha\varphi_\alpha|\varphi_\alpha\rangle|^2$ can be written as

$$\begin{aligned} & |\langle\partial_\alpha\varphi_\alpha|\varphi_\alpha\rangle|^2 \\ = & \left|\langle\psi_0|\frac{(\partial_\alpha\hat{U}_\alpha^\dagger)\sqrt{K_\alpha}-\frac{\partial_\alpha K_\alpha}{2\sqrt{K_\alpha}}\hat{U}_\alpha^\dagger}{K_\alpha}\frac{\hat{U}_\alpha}{\sqrt{K_\alpha}}|\psi_0\rangle\right|^2=\left|\langle\psi_0|\frac{(\partial_\alpha\hat{U}_\alpha^\dagger)\hat{U}_\alpha}{K_\alpha}|\psi_0\rangle-\frac{\partial_\alpha K_\alpha}{2K_\alpha}\right|^2 \\ = & \frac{\langle\psi_0|(\partial_\alpha\hat{U}_\alpha^\dagger)\hat{U}_\alpha|\psi_0\rangle\langle\psi_0|\hat{U}_\alpha^\dagger(\partial_\alpha\hat{U}_\alpha)|\psi_0\rangle}{K_\alpha^2}-\frac{\partial_\alpha K_\alpha}{2K_\alpha^2}\langle\psi_0|[\hat{U}_\alpha^\dagger(\partial_\alpha\hat{U}_\alpha)+(\partial_\alpha\hat{U}_\alpha^\dagger)\hat{U}_\alpha]|\psi_0\rangle \\ & +\frac{(\partial_\alpha K_\alpha)^2}{4K_\alpha^2}.\end{aligned} \tag{S5}$$

Collecting this two results, we obtain

$$\begin{aligned}\mathcal{F}_\alpha &= 4(\langle\partial_\alpha\varphi_\alpha|\partial_\alpha\varphi_\alpha\rangle-|\langle\partial_\alpha\varphi_\alpha|\varphi_\alpha\rangle|^2) \\ &= \frac{\langle\psi_0|(\partial_\alpha\hat{U}_\alpha^\dagger)(\partial_\alpha\hat{U}_\alpha)|\psi_0\rangle}{K_\alpha}-\frac{\langle\psi_0|(\partial_\alpha\hat{U}_\alpha^\dagger)\hat{U}_\alpha|\psi_0\rangle\langle\psi_0|\hat{U}_\alpha^\dagger(\partial_\alpha\hat{U}_\alpha)|\psi_0\rangle}{K_\alpha^2} \\ &= \frac{\langle\psi_\alpha|(\hat{U}_\alpha^\dagger)^{-1}(\partial_\alpha\hat{U}_\alpha^\dagger)(\partial_\alpha\hat{U}_\alpha)\hat{U}_\alpha^{-1}|\psi_\alpha\rangle}{K_\alpha}-\frac{\langle\psi_\alpha|(\hat{U}_\alpha^\dagger)^{-1}(\partial_\alpha\hat{U}_\alpha^\dagger)|\psi_\alpha\rangle\langle\psi_\alpha|(\partial_\alpha\hat{U}_\alpha)\hat{U}_\alpha^{-1}|\psi_\alpha\rangle}{K_\alpha^2} \\ &= \langle\varphi_\alpha|(\hat{U}_\alpha^\dagger)^{-1}(\partial_\alpha\hat{U}_\alpha^\dagger)(\partial_\alpha\hat{U}_\alpha)\hat{U}_\alpha^{-1}|\varphi_\alpha\rangle-\langle\varphi_\alpha|(\hat{U}_\alpha^\dagger)^{-1}(\partial_\alpha\hat{U}_\alpha^\dagger)|\varphi_\alpha\rangle\langle\varphi_\alpha|(\partial_\alpha\hat{U}_\alpha)\hat{U}_\alpha^{-1}|\varphi_\alpha\rangle.\end{aligned} \tag{S6}$$

As we discussed above, $\hat{h}=i(\partial_\alpha\hat{U}_\alpha)\hat{U}_\alpha^{-1}$, then $\mathcal{F}_\alpha$ can further written as

$$\mathcal{F}_\alpha=4(\langle\hat{h}^\dagger\hat{h}\rangle_\alpha-\langle\hat{h}^\dagger\rangle_\alpha\langle\hat{h}\rangle_\alpha), \tag{S7}$$

where $\langle\bullet\rangle_\alpha=\langle\varphi_\alpha|\bullet|\varphi_\alpha\rangle$. If the Hamiltonian is multiplicative $\hat{H}=G\theta$, we have $\hat{U}=e^{-iG\theta t}=e^{-iG\alpha}$, where θ is the parameter which we aim to estimate and $\alpha=\theta t$. If the evolution

time t is a constant then estimating α is equivalent to estimate θ, Eq. (S7) return the form $\mathcal{F}_\alpha = 4(\langle G^\dagger G\rangle_\alpha - \langle G^\dagger\rangle_\alpha\langle G\rangle_\alpha)$ (*63*).

Clearly, the key to QFI is the local generator $\hat{h}$ of the parametric translation of $\hat{U}_\alpha$. In general, it is difficult to derive $\hat{h}$, one will find that $\partial_\alpha \hat{H}_\alpha$ does not commute with $\hat{U}_\alpha$ when calculate $\partial_\alpha \hat{U}_\alpha$. Fortunately, this problem has been discussed in detail already (*80*), the generator $\hat{h}$ can be expressed as (*61*)

$$\hat{h} = i(\partial_\alpha \hat{U}_\alpha)\hat{U}_\alpha^{-1} = \int_0^t e^{-i\mu H_\alpha}\partial_\alpha H_\alpha e^{i\mu H_\alpha} d\mu. \tag{S8}$$

Notably, QFI represents the ultimate precision for a single measurement. However, in non-Hermitian systems, there is gains and losses during the non-unitary evolutions. Relying solely on QFI is insufficient to characterize the ultimate estimation precision when considering fixed resources of probe states. Considering the effect of gains and losses on precision, we multiply the QFI by the normalization coefficient K_α of the output state

$$I_\alpha = K_\alpha \mathcal{F}_\alpha. \tag{S9}$$

This quantity actually characterized the estimation precision for evolution $\hat{U}_\alpha$ in the case of fixed resources of probe states.

Here, we further discuss the precision scaling of a $\mathcal{PT}$-symmetric system $\hat{H}_{PT}$ which is embedded into a larger Hermitian system (*62*,*75*). It is well-known that some $\mathcal{PT}$-symmetric Hamiltonians are pseudo-Hermitian, we can find an invertible Hermitian operator $\hat{\eta}$ that satisfies $\hat{\eta}\hat{H}_{PT} = \hat{H}_{PT}^\dagger\hat{\eta}$. Here, we define $c := \sum_{i=1}^N 1/\lambda_i$ and $\hat{\zeta} = c\hat{\eta} - \hat{I}$, where λ_i represents the ith eigenvalue of $\hat{\eta}$ and $\hat{I}$ is the identity operator. We consider the case of $\mathcal{PT}$-symmetry unbroken, so $\hat{\eta}$ and $\hat{\zeta}$ are positive. The two-qubit dilation Hermitian system can be written as (*62*,*75*)

$$\hat{H}_{tot} = \hat{I} \otimes \hat{H}_s + \hat{\sigma}_y \otimes \hat{V}, \tag{S10}$$

where $\hat{H}_s$ and $\hat{V}$ are Hermitian and satisfy

$$\hat{H}_s - i\hat{V}\hat{\zeta}^{1/2} = \hat{H}_{PT}, \quad \hat{H}_s + i\hat{V}\hat{\zeta}^{-1/2} = \hat{\zeta}^{1/2}\hat{H}_{PT}\hat{\zeta}^{-1/2}. \tag{S11}$$

Solving the above equation, we obtain

$$\hat{H}_s = (\hat{H}_{PT}\hat{\zeta}^{-1/2} + \hat{\zeta}^{1/2}\hat{H}_{PT})(\hat{\zeta}^{1/2} + \hat{\zeta}^{-1/2})^{-1}, \tag{S12}$$

$$\hat{V} = i(\hat{H}_{PT} - \hat{\zeta}^{1/2}\hat{H}_{PT}\hat{\zeta}^{-1/2})(\hat{\zeta}^{1/2} + \hat{\zeta}^{-1/2})^{-1}. \tag{S13}$$

An entangled state $|\Psi_{tot}(t)\rangle$ in two-qubit dilation Hermitian system can be written as

$$|\Psi_{tot}(t)\rangle = |0\rangle \otimes |\psi_{PT}(t)\rangle + |1\rangle \otimes \big(\zeta^{1/2}|\psi_{PT}(t)\rangle\big), \tag{S14}$$

where $|\psi_{PT}(t)\rangle = e^{-i\hat{H}_{PT}t}|\psi_0\rangle$ is the state evolves in $\mathcal{PT}$-symmetric system and $|\psi_0\rangle$ is the initial state of $|\psi_{PT}(t)\rangle$. It can be proved that $|\Psi_{tot}(t)\rangle$ satisfies the Schördinger equation of $\hat{H}_{tot}$, we have

$$\begin{aligned} i\frac{d}{dt}|\Psi_{tot}(t)\rangle &= i\big[|0\rangle \otimes \frac{d}{dt}|\psi_{PT}(t)\rangle + |1\rangle \otimes (\zeta^{1/2}\frac{d}{dt}|\psi_{PT}(t)\rangle)\big] \\ &= |0\rangle \otimes \hat{H}_{PT}|\psi_{PT}(t)\rangle + |1\rangle \otimes (\zeta^{1/2}\hat{H}_{PT}|\psi_{PT}(t)\rangle) \\ &= |0\rangle \otimes \big(\hat{H}_s - i\hat{V}\hat{\zeta}^{1/2}\big)|\psi_{PT}(t)\rangle + |1\rangle \otimes (\hat{H}_s + i\hat{V}\hat{\zeta}^{-1/2})(\hat{\zeta}^{1/2}|\psi_{PT}(t)\rangle) \\ &= \Big[\hat{I} \otimes \hat{H}_s + \hat{\sigma}_y \otimes \hat{V}\Big]\Big[|0\rangle \otimes |\psi_{PT}(t)\rangle + |1\rangle \otimes (\zeta^{1/2}|\psi_{PT}(t)\rangle)\Big] \\ &= \hat{H}_{tot}|\Psi_{tot}(t)\rangle. \end{aligned} \tag{S15}$$

Noticing that the $\hat{\eta}$ inner product $\langle\psi_{PT}(t)|\hat{\eta}|\psi_{PT}(t)\rangle = \langle\psi_0|\hat{\eta}|\psi_0\rangle$ is invariant under the $\mathcal{PT}$ dynamics, so the norm of $|\Psi_{tot}(t)\rangle$ is also invariant $\langle\Psi_{tot}(t)|\Psi_{tot}(t)\rangle = c\langle\psi_0|\hat{\eta}|\psi_0\rangle$. And the normalized state of $|\Psi_{tot}(t)\rangle$ can be written as $|\psi_{tot}(t)\rangle = |\Psi_{tot}(t)\rangle/\sqrt{c\langle\psi_0|\hat{\eta}|\psi_0\rangle}$. Therefore, we can obtain $|\psi_{PT}(t)\rangle/\sqrt{c\langle\psi_0|\hat{\eta}|\psi_0\rangle}$ by performing a projective operator $\hat{P}_0 \otimes \hat{I} = |0\rangle\langle 0| \otimes \hat{I}$ on an entangled state $|\psi_{tot}(t)\rangle$ which evolves in a larger Hermitian system. In other words, we realize the evolution $\hat{U}'_{PT} = \hat{U}_{PT}/\sqrt{c\langle\psi_0|\hat{\eta}|\psi_0\rangle}$ of $\mathcal{PT}$-symmetric system $\hat{H}_{PT}$ in a larger Hermitian system via post-selection.

As we discussed in the main text, multiplying the evolution operator with a scalar function does not change $\mathcal{F}_\alpha$, which is the QFI calculated from $\hat{U}_{PT}$. However, for fixed resource of probe states, the scalar function characterizes the probability of realizing $\hat{U}_{PT}$, which will affect the ultimate estimation precision. Therefore, the ultimate estimation precision is actually limited by $K_\alpha \mathcal{F}_\alpha / \big(c\langle\psi_0|\hat{\eta}|\psi_0\rangle\big)$. The coefficient $c\langle\psi_0|\hat{\eta}|\psi_0\rangle$ is independent of time t. Obviously, the estimation precision scaling for the evolution $\hat{U}_{PT}$ is the same as that for the evolution $\hat{U}'_{PT}$.

B. Analysis of QFI for two level non-Hermitian Hamiltonians near EP

Consider a two-level non-Hermitian operator $\hat{O}$ with two nondegenerate eigenvalues $\lambda_{1,2}$ and two linearly independent eigenvectors $|\lambda_{1,2}\rangle$. Suppose we have an arbitrary normalized state which is able to be expressed by the superposition of eigenstates

$$|\psi\rangle = a|\lambda_1\rangle + b|\lambda_2\rangle, \tag{S16}$$

where a and b are complex numbers, then we define the variance of non-Hermitian operator $\hat{O}$ as $(\Delta\hat{O})^2 = \langle\psi|\hat{O}^\dagger\hat{O}|\psi\rangle - \langle\psi|\hat{O}^\dagger|\psi\rangle\langle\psi|\hat{O}|\psi\rangle$. We can further rewrite it as follow

$$(\Delta\hat{O})^2 = \langle\psi|\hat{O}^\dagger\hat{O}|\psi\rangle - \langle\psi|\hat{O}^\dagger|\psi\rangle\langle\psi|\hat{O}|\psi\rangle = \langle\psi|\hat{O}^\dagger(I - |\psi\rangle\langle\psi|)\hat{O}|\psi\rangle = \langle\psi|\hat{O}^\dagger\hat{P}\hat{O}|\psi\rangle. \tag{S17}$$

We can see that for two-level systems, the projective operator $\hat{P} = I - |\psi\rangle\langle\psi| = |\phi\rangle\langle\phi|$ is exactly the pure state that orthogonal to $|\psi\rangle$. Assume $|\phi\rangle = c|\lambda_1\rangle + d|\lambda_2\rangle$, then we have

$$\langle\phi|\psi\rangle = ac^* + bd^* + ad^*\langle\lambda_2|\lambda_1\rangle + bc^*\langle\lambda_1|\lambda_2\rangle = 0, \tag{S18}$$

and $\hat{O}|\psi\rangle$ can be written as $\hat{O}|\psi\rangle = a\lambda_1|\lambda_1\rangle + b\lambda_2|\lambda_2\rangle$. Therefore, the variance can be further written as

$$(\Delta\hat{O})^2 = \langle\psi|\hat{O}^\dagger\hat{P}\hat{O}|\psi\rangle = |\langle\phi|\hat{O}|\psi\rangle|^2 = \left|ac^*\lambda_1 + bd^*\lambda_2 + ad^*\lambda_1\langle\lambda_2|\lambda_1\rangle + bc^*\lambda_2\langle\lambda_1|\lambda_2\rangle\right|^2. \tag{S19}$$

According to Eq. (S18), we have

$$
\begin{aligned}
(\Delta\hat{O})^2 &= \big|ac^*\lambda_1 + bd^*\lambda_2 + ad^*\lambda_1\langle\lambda_2|\lambda_1\rangle - \lambda_2\big(ac^* + bd^* + ad^*\langle\lambda_2|\lambda_1\rangle\big)\big|^2 \\
&= \big|ac^*(\lambda_1 - \lambda_2) + ad^*\langle\lambda_2|\lambda_1\rangle(\lambda_1 - \lambda_2)\big|^2 \\
&= \big|a\big|^2 \cdot \big|(\lambda_1 - \lambda_2)\big|^2 \cdot \big|c^* + d^*\langle\lambda_2|\lambda_1\rangle\big|^2. \qquad \text{(S20)}
\end{aligned}
$$

Obviously, for a given state $|\psi\rangle = a|\lambda_1\rangle + b|\lambda_2\rangle$, the variance $(\Delta\hat{O})^2$ is determined by the modulus of difference between two eigenvalues λ_1 and λ_2. As we discussed in section A, for a multiplicative pseudo-Hermitian Hamiltonian $\hat{H} = Gs$, the QFI is the variance of the G which has the same properties with $\hat{H}$. It is well-known that for pseudo-Hermitian Hamiltonians, the eigenvalues gradually degenerate as EP is approached. Therefore, for a given state $|\psi\rangle = a|\lambda_1\rangle + b|\lambda_2\rangle$, the QFI of multiplicative pseudo-Hermitian Hamiltonian $\hat{H}$ would tends to zero near EP.

However, as for non-multiplicative Hamiltonians, QFI is the variance of the generator h, the properties of h near EP could be much different from that of Hamiltonian. For example, consider a non-Hermitian Hamiltonian $\hat{H}_1 = I\sin\alpha\sigma_z + \cos\alpha\sigma_x$, α is the unknown parameter, the evolution operator is $U_1 = e^{-iH_1 t}$. Then we can obtain the eigenvalues of local generator h_1

$$\lambda_1 = -\sec 2\alpha\sqrt{\frac{\cos(2t\sqrt{\cos 2\alpha}) + t^2\sin 2\alpha\sin 4\alpha - 1}{2}}, \qquad \text{(S21)}$$

$$\lambda_2 = \sec 2\alpha\sqrt{\frac{\cos(2t\sqrt{\cos 2\alpha}) + t^2\sin 2\alpha\sin 4\alpha - 1}{2}}. \qquad \text{(S22)}$$

In the case of $\mathcal{PT}$-symmetry unbroken, if $t \gg 1$, the modulus of difference $|\Delta\lambda| = |\lambda_2 - \lambda_1|$ increases as EP ($\alpha = \pi/4$) is approached. Thus, the QFI of non-multiplicative Hamiltonians could increase near EP. Noticed that the above discussion applies to the case that EP is not exactly reached. Because two eigenstates would coalesce at EP, the state $|\psi\rangle = a|\lambda_1\rangle + b|\lambda_2\rangle$ cannot be able to represent an arbitrary state anymore, it is just the eigenstate.

Furthermore, we are also able to analyse that whether we can achieve Heisenberg precision for multiplicative non-Hermitian Hamiltonians. For example, the generator of the parameter α for general time t of the non-Hermitian Hamiltonian $\hat{H}_{PT}(\alpha)$ in the main text is

$$\hat{h}_\alpha(t) = \frac{\sec\alpha}{2}\begin{pmatrix} i[\sec\alpha + \sin(2st\cos\alpha) - 2st\sin^2\alpha] & \sec\alpha\cos(\alpha - 2st\cos\alpha) - 2st\sin\alpha - 1 \\ \sec\alpha\cos(\alpha + 2st\cos\alpha) + 2st\sin\alpha - 1 & -i[\sec\alpha + \sin(2st\cos\alpha) - 2st\sin^2\alpha] \end{pmatrix}, \tag{S23}$$

and we can obtain the eigenvalues of $\hat{h}_\alpha(t)$ as follows

$$\lambda_\pm = \pm\frac{\sec\alpha\sqrt{4\cos(2st\cos\alpha) - 4 + s^2t^2(1-\cos 4\alpha)}}{2\sqrt{2}}. \tag{S24}$$

As shown in Fig. S1, the growth of the modulus of difference $|\lambda_+ - \lambda_-|$ reaches the scale of t. According to Eq. (S20), we can derive that the growth of of QFI reaches t^2, i.e., Heisenburg limit, and we obtain the exact QFI for general time t when probe state is $|\psi_0\rangle = |0\rangle$ as follows

$$\mathcal{F}_\alpha(t) = \Big[\frac{1 - \sec\alpha\cos(\alpha - 2st\cos\alpha) + 2st\sin\alpha}{\sec\alpha - \sin(\alpha - 2st\cos\alpha)\tan\alpha}\Big]^2. \tag{S25}$$

Obviously, there is t^2 in $\mathcal{F}_\alpha(t)$.

C. Proof of the condition for optimal measurements

Based on the expression of QFI we proposed, we give the detailed proof of the condition for optimal measurement in this section. Consider an arbitrary Hermitian operator $\hat{A}$ as measurement operator. According to the error-propagation formula (*65,66*), we have

$$(\Delta\alpha)^2 = \frac{(\Delta\hat{A})^2}{n|\partial_\alpha\langle\hat{A}\rangle_\alpha|^2} = \frac{(\Delta\hat{A})^2}{n|\langle\partial_\alpha\varphi_\alpha|\hat{A}|\varphi_\alpha\rangle + \langle\varphi_\alpha|\hat{A}|\partial_\alpha\varphi_\alpha\rangle|^2}, \tag{S26}$$

where $|\varphi_\alpha\rangle$ is the normalized output state after evolution. For simplicity, let $Q = \langle\partial_\alpha\varphi_\alpha|\hat{A}|\varphi_\alpha\rangle + \langle\varphi_\alpha|\hat{A}|\partial_\alpha\varphi_\alpha\rangle$ and expand it as follow

$$\begin{aligned} Q &= \langle\partial_\alpha\varphi_\alpha|\hat{A}|\varphi_\alpha\rangle + \langle\varphi_\alpha|\hat{A}|\partial_\alpha\varphi_\alpha\rangle = \langle\psi_0|\partial_\alpha\Big(\frac{\hat{U}_\alpha^\dagger}{\sqrt{K_\alpha}}\Big)\hat{A}|\varphi_\alpha\rangle + \langle\varphi_\alpha|\hat{A}\partial_\alpha\Big(\frac{\hat{U}_\alpha}{\sqrt{K_\alpha}}\Big)|\psi_0\rangle \\ &= \langle\psi_0|\frac{(\partial_\alpha\hat{U}_\alpha^\dagger)\sqrt{K_\alpha} - \frac{\partial_\alpha K_\alpha}{2\sqrt{K_\alpha}}\hat{U}_\alpha^\dagger}{K_\alpha}\hat{A}|\varphi_\alpha\rangle + \langle\varphi_\alpha|\hat{A}\frac{(\partial_\alpha\hat{U}_\alpha)\sqrt{K_\alpha} - \frac{\partial_\alpha K_\alpha}{2\sqrt{K_\alpha}}\hat{U}_\alpha}{K_\alpha}|\psi_0\rangle \\ &= \langle\psi_0|\frac{\partial_\alpha\hat{U}_\alpha^\dagger}{\sqrt{K_\alpha}}\hat{A}|\varphi_\alpha\rangle + \langle\varphi_\alpha|\hat{A}\frac{\partial_\alpha\hat{U}_\alpha}{\sqrt{K_\alpha}}|\psi_0\rangle - \frac{\partial_\alpha K_\alpha}{K_\alpha}\langle\hat{A}\rangle_\alpha \\ &= i[\langle\varphi_\alpha|(-i)(\hat{U}_\alpha^\dagger)^{-1}(\partial_\alpha\hat{U}_\alpha^\dagger)\hat{A}|\varphi_\alpha\rangle - \langle\varphi_\alpha|i\hat{A}(\partial_\alpha\hat{U}_\alpha)\hat{U}_\alpha^{-1}|\varphi_\alpha\rangle] - \frac{\partial_\alpha K_\alpha}{K_\alpha}\langle\hat{A}\rangle_\alpha \\ &= i(\langle\hat{h}^\dagger\hat{A}\rangle_\alpha - \langle\hat{A}\hat{h}\rangle_\alpha) - \frac{\partial_\alpha K_\alpha}{K_\alpha}\langle\hat{A}\rangle_\alpha, \end{aligned} \tag{S27}$$

where $K_\alpha = \langle\psi_0|\hat{U}_\alpha^\dagger \hat{U}_\alpha|\psi_0\rangle = \langle\psi_\alpha|\psi_\alpha\rangle$ is the normalized coefficient, then $(\partial_\alpha K_\alpha)/K_\alpha$ can be further written as

$$\begin{aligned}\frac{\partial_\alpha K_\alpha}{K_\alpha} &= \frac{\langle\psi_0|(\partial_\alpha \hat{U}_\alpha^\dagger)\hat{U}_\alpha|\psi_0\rangle + \langle\psi_0|\hat{U}_\alpha^\dagger(\partial_\alpha \hat{U}_\alpha)|\psi_0\rangle}{K_\alpha} \\ &= \frac{\langle\psi_\alpha|(\hat{U}_\alpha^\dagger)^{-1}(\partial_\alpha \hat{U}_\alpha^\dagger)|\psi_\alpha\rangle + \langle\psi_\alpha|(\partial_\alpha \hat{U}_\alpha)\hat{U}_\alpha^{-1}|\psi_\alpha\rangle}{K_\alpha} \\ &= i(\langle\hat{h}^\dagger\rangle_\alpha - \langle\hat{h}\rangle_\alpha). \qquad \text{(S28)}\end{aligned}$$

Substitute this result into Eq. (S27), we obtain

$$\begin{aligned}|Q|^2 &= |(\langle\hat{h}^\dagger\hat{A}\rangle_\alpha - \langle\hat{A}\hat{h}\rangle_\alpha) - (\langle\hat{h}^\dagger\rangle_\alpha - \langle\hat{h}\rangle_\alpha)\langle\hat{A}\rangle_\alpha|^2 \\ &= -(\langle\hat{h}^\dagger\hat{A}\rangle_\alpha - \langle\hat{A}\hat{h}\rangle_\alpha)^2 - (\langle\hat{h}^\dagger\rangle_\alpha - \langle\hat{h}\rangle_\alpha)^2\langle\hat{A}\rangle_\alpha^2 + 2(\langle\hat{h}^\dagger\rangle_\alpha - \langle\hat{h}\rangle_\alpha)(\langle\hat{h}^\dagger\hat{A}\rangle_\alpha - \langle\hat{A}\hat{h}\rangle_\alpha)\langle\hat{A}\rangle_\alpha \\ &= -(\langle\hat{h}^\dagger\hat{A}\rangle_\alpha + \langle\hat{A}\hat{h}\rangle_\alpha)^2 - (\langle\hat{h}^\dagger\rangle_\alpha + \langle\hat{h}\rangle_\alpha)^2\langle\hat{A}\rangle_\alpha^2 + 2(\langle\hat{h}^\dagger\rangle_\alpha - \langle\hat{h}\rangle_\alpha)(\langle\hat{h}^\dagger\hat{A}\rangle_\alpha - \langle\hat{A}\hat{h}\rangle_\alpha)\langle\hat{A}\rangle_\alpha \\ &\quad +4\big[\langle\hat{h}^\dagger\hat{A}\rangle_\alpha\langle\hat{A}\hat{h}\rangle_\alpha + \langle\hat{h}^\dagger\rangle_\alpha\langle\hat{h}\rangle_\alpha\langle\hat{A}\rangle_\alpha^2\big] \\ &= -(\langle\hat{h}^\dagger\hat{A}\rangle_\alpha + \langle\hat{A}\hat{h}\rangle_\alpha)^2 - (\langle\hat{h}^\dagger\rangle_\alpha + \langle\hat{h}\rangle_\alpha)^2\langle\hat{A}\rangle_\alpha^2 + 2(\langle\hat{h}^\dagger\rangle_\alpha + \langle\hat{h}\rangle_\alpha)(\langle\hat{h}^\dagger\hat{A}\rangle_\alpha + \langle\hat{A}\hat{h}\rangle_\alpha)\langle\hat{A}\rangle_\alpha \\ &\quad +4\big[\langle\hat{h}^\dagger\hat{A}\rangle_\alpha\langle\hat{A}\hat{h}\rangle_\alpha + \langle\hat{h}^\dagger\rangle_\alpha\langle\hat{h}\rangle_\alpha\langle\hat{A}\rangle_\alpha^2 - \langle\hat{h}^\dagger\hat{A}\rangle_\alpha\langle\hat{h}\rangle_\alpha\langle\hat{A}\rangle_\alpha - \langle\hat{A}\hat{h}\rangle_\alpha\langle\hat{h}^\dagger\rangle_\alpha\langle\hat{A}\rangle_\alpha\big]. \qquad \text{(S29)}\end{aligned}$$

Here, we define an operator $\delta\hat{M} = \hat{M} - \langle\hat{M}\rangle_\alpha$, the variance of $\hat{M}$ over $|\varphi_\alpha\rangle$ is defined by

$$(\Delta\hat{M})^2 = \langle\varphi_\alpha|(\hat{M} - \langle M\rangle_\alpha)^\dagger(\hat{M} - \langle M\rangle_\alpha)|\varphi_\alpha\rangle = \langle\varphi_\alpha|\delta\hat{M}^\dagger\delta\hat{M}|\varphi_\alpha\rangle. \qquad \text{(S30)}$$

According to the non-Hermitian uncertainty relationship $(\Delta\hat{A})^2(\Delta\hat{B})^2 \geq |\langle\hat{A}^\dagger\hat{B}\rangle - \langle\hat{A}^\dagger\rangle\langle\hat{B}\rangle|^2$ (*67–71*), we have

$$\begin{aligned}(\Delta\hat{h})^2(\Delta\hat{A})^2 &\geq |\langle\hat{h}^\dagger\hat{A}\rangle_\alpha - \langle\hat{h}^\dagger\rangle\langle\hat{A}\rangle_\alpha|^2 \\ &= \langle\hat{h}^\dagger\hat{A}\rangle_\alpha\langle\hat{A}\hat{h}\rangle_\alpha + \langle\hat{h}^\dagger\rangle_\alpha\langle\hat{h}\rangle_\alpha\langle\hat{A}\rangle_\alpha^2 - \langle\hat{h}^\dagger\hat{A}\rangle_\alpha\langle\hat{h}\rangle_\alpha\langle\hat{A}\rangle_\alpha - \langle\hat{A}\hat{h}\rangle_\alpha\langle\hat{h}^\dagger\rangle_\alpha\langle\hat{A}\rangle_\alpha. \qquad \text{(S31)}\end{aligned}$$

Obviously, the expression in the last brackets of Eq. (S29) is the same as the result in Eq. (S31), so we have

$$\begin{aligned}|Q|^2 &\leq 4(\Delta\hat{h})^2(\Delta\hat{A})^2 - (\langle\hat{h}^\dagger\hat{A}\rangle_\alpha + \langle\hat{A}\hat{h}\rangle_\alpha)^2 - (\langle\hat{h}^\dagger\rangle_\alpha + \langle\hat{h}\rangle_\alpha)^2\langle\hat{A}\rangle_\alpha^2 \\ &\quad +2(\langle\hat{h}^\dagger\rangle_\alpha + \langle\hat{h}\rangle_\alpha)(\langle\hat{h}^\dagger\hat{A}\rangle_\alpha + \langle\hat{A}\hat{h}\rangle_\alpha)\langle\hat{A}\rangle_\alpha \\ &\leq 4(\Delta\hat{h})^2(\Delta\hat{A})^2 - \big[(\langle\hat{h}^\dagger\hat{A}\rangle_\alpha + \langle\hat{A}\hat{h}\rangle_\alpha) - (\langle\hat{h}^\dagger\rangle_\alpha + \langle\hat{h}\rangle_\alpha)\langle\hat{A}\rangle_\alpha\big]^2. \qquad \text{(S32)}\end{aligned}$$

Define $|f\rangle = \delta\hat{h}|\varphi_\alpha\rangle$ and $|g\rangle = \delta\hat{A}|\varphi_\alpha\rangle$, we have $\langle f|g\rangle = \langle\hat{h}^\dagger\hat{A}\rangle_\alpha - \langle\hat{h}_\alpha^\dagger\rangle\langle\hat{A}\rangle_\alpha$. Then, we further obtain the inequality as follow

$$\begin{aligned}|Q|^2 &\leq 4(\Delta\hat{h})^2(\Delta\hat{A})^2 - \big[(\langle\hat{h}^\dagger\hat{A}\rangle_\alpha - \langle\hat{h}^\dagger\rangle_\alpha\langle\hat{A}\rangle_\alpha) + (\langle\hat{A}\hat{h}\rangle_\alpha - \langle\hat{h}\rangle_\alpha\langle\hat{A}\rangle_\alpha)\big]^2 \\ &= 4(\Delta\hat{h})^2(\Delta\hat{A})^2 - (\langle f|g\rangle + \langle g|f\rangle) \\ &\leq 4(\Delta\hat{h})^2(\Delta\hat{A})^2. \qquad \text{(S33)}\end{aligned}$$

According to Cauchy-Schwarz inequality, the first inequality is saturated when $|f\rangle$ and $|g\rangle$ is linearly related, i.e., $|f\rangle = c|g\rangle$. And the second inequality is further saturated if c is an imaginary number. Therefore, we could derive the inequality

$$(\Delta\alpha)^2 = \frac{(\Delta\hat{A})^2}{n|\partial_\alpha\langle\hat{A}\rangle_\alpha|^2} = \frac{(\Delta\hat{A})^2}{n|Q|^2} \geq \frac{(\Delta\hat{A})}{4n(\Delta\hat{h})^2(\Delta\hat{A})^2} = \frac{1}{n\mathcal{F}_\alpha}, \tag{S34}$$

it is exactly the QCRB, as we discussed above, it is saturated when

$$|f\rangle = ic|g\rangle, \tag{S35}$$

where c is a real number.

D. The reason for normalization

It is well-known that the probability may not conserve in non-Hermitian systems evolution. However, for measurement process, the probabilities of measurement outcomes always sum up to 1, even if there are losses or gains during evolution process, and the final state is normalized by dividing its trace. As shown in Fig. S2, we use the polarization of photons to encode states, where horizontally polarized state $|H\rangle$ is encoded as $|0\rangle$, and vertically polarized state $|V\rangle$ is encoded as $|1\rangle$, the photons are prepared as initial state $\rho_0 = |\psi_0\rangle\langle\psi_0|$ and then evolves in the non-Hermitian quantum system, which will lead to the decay of the total number of photons N_{tot}, i.e., $N = N_{tot}\mathrm{Tr}\{\rho_\theta\} < N_{tot}$. However, the probability is still normalized when we measure the final state $\rho_\theta = U_\theta\rho_0 U_\theta^\dagger$. For instance, we measure the final state with the set of projection operation $\Pi = \sum_i |i\rangle\langle i|$, the probabilities of two measurement outcomes respectively are $P_0 = N_H/N = (N_{tot}\mathrm{Tr}\{\rho_\theta|0\rangle\langle 0|\})/(N_{tot}\mathrm{Tr}\{\rho_\theta\})$ and $P_1 = N_V/N = (N_{tot}\mathrm{Tr}\{\rho_\theta|1\rangle\langle 1|\})/(N_{tot}\mathrm{Tr}\{\rho_\theta\})$, the total probability is $P = P_0 + P_1 = 1$, where N_H and N_V respectively are the number of horizontally polarized photons and vertically polarized photons. For measurement, the final state is actually

$$\widetilde{\rho}_\theta = \rho_\theta/\mathrm{Tr}\{\rho_\theta\}. \tag{S36}$$

Therefore, we normalize the final state by dividing its trace, for pure states, the normalized final state is

$$|\varphi_\theta\rangle = \frac{|\psi_\theta\rangle}{\sqrt{\langle\psi_\theta|\psi_\theta\rangle}}. \tag{S37}$$

II. EXPERIMENT

Before we introduce our experimental setup, we first illustrate the optics elements in our experiment.

1) The Jones matrix of half-wave plate (HWP) and quarter-wave plates (QWP) in our experiment respectively are

$$U_{HWP} = \begin{pmatrix} \cos 2\theta & \sin 2\theta \\ \sin 2\theta & -\cos 2\theta \end{pmatrix}, \quad U_{QWP} = \begin{pmatrix} \cos^2\theta + i\sin^2\theta & (1-i)\cos\theta\sin\theta \\ (1-i)\cos\theta\sin\theta & i\cos^2\theta + \sin^2\theta \end{pmatrix}, \tag{S38}$$

   where θ is the angle between the light polarization direction and the fast axis of the wave plate.

2) The polarization beam splitter (PBS) in our experiment transmits the horizontal polarized photons and reflect the vertical polarized photons, as shown in Fig. S3A.

3) The beam displacer (BD) in our experiment transmits the horizontal polarized photons, but separates the vertical photons into new path which is about 4mm from the original path, as shown in Fig. S3B.

A. Photon-pair source

The central wavelength of pump laser in our experiment is 405 nm. The energy of pump laser is adjust by QWP1, HWP1 and PBS1, meanwhile the state is purified into $|H\rangle$. If the initial state is prepared as $(|H\rangle + |V\rangle)/\sqrt{2}$, this experimental set could generate entangled photon pair $(|HV\rangle + |VH\rangle)/\sqrt{2}$. In our experiment, only the heralded single photon source is needed, so we prepare the initial state as $|H\rangle$. A dichroic mirror (DM) reflect pump light into the triangle sagnac interferometer, and then PPKTP crystal is clockwise pumped. After type-II phase-matched spontaneous parametric down-conversion process, one 405 nm photon splits into two 810 nm photons ($|H\rangle \to |H\rangle_1|V\rangle_2$). The DPBS and two mirrors are affective for both 405 nm and 810 nm photons, but DM does not reflect 810 nm photons. So these two 810nm photons are separated into two paths, then filtered by long-wave path filter (LPF) and collected into single-mode fibers. In our experiment, the power of pumped light is 2 mw, and we could obtain 80000 coincidences per second.

B. Theoretical framework of $\hat{U}_{PT}$ evolution

The non-unitary $\mathcal{PT}$-symmetric evolution $\hat{U}_{PT}$ consists of the probe qubit ($|H,V\rangle$) and ancilla qubit ($|a,b\rangle$), by performing a projective operator on ancilla qubit, we could effectively construct a non-unitary $\mathcal{PT}$-symmetric evolution $\hat{U}'_{PT} = F\hat{U}_{PT}$ (*26*), as shown in Fig. S5. The non-unitary $\mathcal{PT}$-symmetric evolution $\hat{U}_{PT}$ can be written as $\hat{U}_{PT} = |\psi_H\rangle\langle H| + |\psi_V\rangle\langle V|$

($|\psi_H\rangle = \hat{U}_{PT}|H\rangle$, $|\psi_V\rangle = \hat{U}_{PT}|V\rangle$). The operator $\hat{U}_{tot}$ is unitary, but in Fig. S5, we do not concentrate on the photons (a new qubit) losses at H3, H4 and PBS2, the left two-qubit evolution $\hat{U}$ can be expressed as follow,

$$\begin{aligned}\hat{U} &= \gamma(|\psi_H\rangle\langle H|\otimes|a\rangle\langle a| + |\psi_V\rangle\langle V|\otimes|b\rangle\langle a| + |\psi_H^{\perp}\rangle\langle V|\otimes|a\rangle\langle b| + |\psi_V^{\perp}\rangle\langle H|\otimes|b\rangle\langle b|),\\ &= p|\varphi_H\rangle\langle H|\otimes|a\rangle\langle a| + q|\varphi_V\rangle\langle V|\otimes|b\rangle\langle a| + p|\varphi_H^{\perp}\rangle\langle V|\otimes|a\rangle\langle b| + q|\varphi_V^{\perp}\rangle\langle H|\otimes|b\rangle\langle b|,\end{aligned} \tag{S39}$$

where $p = \sin 2(\phi_1 - \phi_2)$ and $q = \cos 2\phi_2$ are controlled by H3 (ϕ_1) and H4 (ϕ_2), $p^2/q^2 = \langle\psi_H|\psi_H\rangle/\langle\psi_V|\psi_V\rangle$, $|\varphi_H\rangle = |\psi_H\rangle/\sqrt{\langle\psi_H|\psi_H\rangle}$, $|\varphi_V\rangle = |\psi_V\rangle/\sqrt{\langle\psi_V|\psi_V\rangle}$ and $\gamma = p/\sqrt{\langle\psi_H|\psi_H\rangle} = q/\sqrt{\langle\psi_V|\psi_V\rangle}$, if $p = q = 1$, $\hat{U} = \hat{U}_{tot}$ is an unitary evolution. As shown in Fig. S6, p and q are controlled by H3, H4 and PBS2, $|\varphi_H\rangle$ and $|\varphi_V\rangle$ are prepared by H5, Q1, H6 and Q1 respectively, $|\varphi_H^{\perp}\rangle$ and $|\varphi_V^{\perp}\rangle$ are the corresponding orthogonal states. After preparing $|\varphi_H\rangle|a\rangle$ and $|\varphi_V\rangle|b\rangle$, BD2 and BD3 combine their horizontal and vertical components into two paths respectively. Then the projective operator is realized by PBS3 and H9 set at $22.5°$, it can be expressed as

$$\hat{P} = I \otimes \frac{|a\rangle + |b\rangle}{\sqrt{2}} \frac{\langle a| + \langle b|}{\sqrt{2}}, \tag{S40}$$

where I is identity operator. The evolution after performing projective operator is

$$\hat{U}'_{PT} = \hat{P}\hat{U} = \frac{p|\varphi_H\rangle\langle H| + q|\varphi_V\rangle\langle V|}{\sqrt{2}} \otimes \frac{|a\rangle + |b\rangle}{\sqrt{2}}\langle a|. \tag{S41}$$

In our experiment, the initial state of ancilla qubit is always $|a\rangle$, then, for an arbitrary initial state $|\psi_0\rangle = \cos 2\phi|H\rangle + \sin 2\phi|V\rangle$ of probe qubit, the final state after evolution is $(p\cos 2\phi|\varphi_H\rangle + q\sin 2\phi|\varphi_V\rangle)/\sqrt{2}$. Thus, the evolution we construct is proportional to the theoretical non-Hermitian system evolution, for probe qubit, we have

$$\hat{U}'_{PT} = \frac{p|\varphi_H\rangle\langle H| + q|\varphi_V\rangle\langle V|}{\sqrt{2}} = \frac{\gamma}{\sqrt{2}}(|\psi_H\rangle\langle H| + |\psi_V\rangle\langle V|) = F\hat{U}_{PT}. \tag{S42}$$

where $F = \gamma/\sqrt{2}$ is a scalar function.

C. Optimal measurements

In this section, we prove that $\hat{A} = |0\rangle\langle 0|$ is optimal measurement for both multiplicative and non-multiplicative Hamiltonians when the probe state is $|0\rangle$. In the case of estimate s, according to the condition for optimal measurements.

$$|f\rangle = i\frac{t\cos\alpha\cos(\alpha - 2st\cos\alpha)\sin\alpha}{\cos^2(a - 2st\cos\alpha) + \sin^2(st\cos\alpha)}|g\rangle, \tag{S43}$$

so the measurement $\hat{A}$ is indeed the optimal measurement when estimate s.

In the case of estimate α, we further experimentally verify the result. We first give the theoretical analysis.t he probe state is prepared as an arbitrary linear polarization pure state $|\psi_0\rangle = \cos 2\phi|0\rangle + \sin 2\phi|1\rangle$, we change the probe state from $|0\rangle$ to $|1\rangle$ ($\phi = 0^\circ \sim 45^\circ$) and perform $\hat{A} = |0\rangle\langle 0|$ as measurement. According to Eq. (S35), we have

$$\begin{aligned} &|f\rangle \\ =\ & \frac{2\cos\alpha[st\sin\alpha(\cos 4\alpha - i\sin 4\phi) + \sin^2(st\cos\alpha)] + (i\sin 4\phi - \cos 4\phi\sin\alpha)\sin(2st\cos\alpha)}{2[\cos 2\phi\cos(\alpha - st\cos\alpha) - i\sin 2\phi\sin(st\cos\alpha)][\cos(\alpha + st\cos\alpha)\sin 2\phi - i\cos 2\phi\sin(st\cos\alpha)]}|g\rangle, \end{aligned} \tag{S44}$$

we can see that the condition for optimal measurement is satisfied only if $\phi = k\pi/4$, i.e., the probe state is $|0\rangle$ or $|1\rangle$.

In our experiment, we set that $s = 1$, $\alpha = \pi/10$ and $t = \pi/[2s\cos(\pi/10)]$. To get the statistic of the estimation, we make 5000 maximum likelihood estimates and get the distribution of estimators $\hat{\alpha}$, the experimental results is shown in Fig. S7. Due to the fluctuation of the data, the estimator $\hat{\alpha}$ cannot be calculated by maximum likelihood estimation when $\phi = 22.5^\circ$, $\phi = 27^\circ$ and $\phi = 31.5^\circ$, so the corresponding deviations are not plotted. As shown in Fig. S7B, the standard deviation of estimator reaches QCRB when the probe state is $|0\rangle$ and $|1\rangle$, it is consistent with our theoretical analysis. We also give the exact data of Fig. S7, as shown in Table. S1 and Table. S2.

D. Maximum likelihood estimation

When we estimate the parameter with different probe states, we apply maximum likelihood estimation to get the estimator $\hat{\alpha}$. For two-level systems, the distribution of measurement outcomes obeys binomial distribution, for n independent measurements, the likelihood function is

$$L(n, x|\alpha) = \frac{n!}{(n-x)!x!}p(\alpha)^x[1 - p(\alpha)]^{(n-x)}. \tag{S45}$$

this expression represents the probability that there are x measurement outcomes $|0\rangle$ in n measurements, and $p(\alpha) = |\langle 0|\varphi_\alpha\rangle|^2$ is the probability of obtaining $|0\rangle$. The logarithmic of likelihood function is

$$\ln[L(n, x|\alpha)] = \ln[\frac{n!}{(n-x)!x!}] + x\ln[p(\alpha)] + (n-x)\ln[1 - p(\alpha)]. \tag{S46}$$

Then we solve the differential equation

$$0 = \frac{d\ln[L(n, x|\alpha)]}{d\alpha} = x\frac{d\ln[p(\alpha)]}{d\alpha} + (n-x)\frac{d\ln[1 - p(\alpha)]}{d\alpha}. \tag{S47}$$

The solution of the equation is the estimator $\hat{\alpha} = \hat{\alpha}(n, x)$, it is the function of n and x. Substituting the experimental results into $\hat{\alpha}(n, x)$, we obtain the estimate of the parameter α. And we further obtain the distribution of estimation by repeating this process.

E. Experimental datas of reaching Heisenberg scaling

In the main text, we show the standard deviation of estimation results and a part of distribution of estimators, here we present more detailed results. As shown in Table. S3 and Table. S4, we give the exact experimental and theoretical results for different time points. In Table. S3 and Table. S4, we can see that the average of estimator $\hat{s}$ and $\hat{\alpha}$ is slightly different from the theoretical value $s = 1$ and $\alpha = \pi/4$, because of the error of the non-unitary $\mathcal{PT}$-symmetric evolution $\hat{U}'_{PT}$ we constructed. As show in Fig. S8 and Table. S5, we can see that although the errors of the probabilities we measured is quite small, there are still errors between the average of estimators and theoretical value, especially for the probe states that lead to worse estimation precision.

F. Non-Hermitian Hamiltonian without $\mathcal{PT}$ or anti-$\mathcal{PT}$ symmetry

To demonstrate the generality of our theory, we consider a Hamiltonian without any special symmetries given by

$$\hat{H}_\kappa = \begin{pmatrix} 0 & \kappa \\ 1 & 0 \end{pmatrix}, \tag{S48}$$

where κ is the unknown real parameter and $\kappa \neq 1$. It can be seen that $\hat{H}_\kappa$ is neither $\mathcal{PT}$-symmetric nor anti $\mathcal{PT}$-symmetric. The corresponding evolution operator $\hat{U}_\kappa$ can be deduced from the Hamiltonian $\hat{H}_\kappa$ as

$$\hat{U}_\kappa(t) = \begin{pmatrix} \cos(t\sqrt{\kappa}) & -i\sqrt{\kappa}\sin(t\sqrt{\kappa}) \\ \frac{-i}{\sqrt{\kappa}}\sin(t\sqrt{\kappa}) & \cos(t\sqrt{\kappa}) \end{pmatrix}. \tag{S49}$$

According to Eq. (S2), we can obtain the generator as

$$\hat{h}_\kappa(t) = \frac{1}{4\kappa\sqrt{\kappa}} \begin{pmatrix} i2\sqrt{\kappa}\sin^2(t\sqrt{\kappa}) & 2t\kappa\sqrt{\kappa} + \kappa\sin(2t\sqrt{\kappa}) \\ 2t\sqrt{\kappa} - \sin(2t\sqrt{\kappa}) & -i2\sqrt{\kappa}\sin^2(t\sqrt{\kappa}) \end{pmatrix}. \tag{S50}$$

The eigenvalues of $\hat{h}_\kappa(t)$ are $\lambda_\pm = \pm\sqrt{[-1 + 2\kappa t^2 + \cos(2t\sqrt{\kappa})]/(8\kappa)}$. As shown in Fig.S9, the growth of the modulus of the difference, $|\Delta\lambda| = |\lambda_+ - \lambda_-|$, reaches the scale of t, indicating that the growth of the quantum Fisher information scales as t^2. The initial probe state is prepared as $|\psi_0\rangle = |0\rangle$. By utilizing Eq. (S7), we can calculate the QFI as

$$\mathcal{F}_\kappa(t) = \frac{[-2t\sqrt{\kappa} + \sin(2t\sqrt{\kappa})]^2}{4\kappa[\kappa\cos^2(t\sqrt{\kappa}) + \sin^2(t\sqrt{\kappa})]^2}. \tag{S51}$$

It can be observed that the QFI indeed exhibits a growth scaling of t^2, which represents the Heisenberg scaling.

Using a similar scheme and experimental setup in Fig. S5 and S6, we realize the non-unitary evolution operator $\hat{U}'_\kappa = F\hat{U}_\kappa$, where F is a real scalar. It is important to note that the states $|\varphi'_{H,V}\rangle$ realized in path a and b differ from those in the experiment of $\mathcal{PT}$-symmetry Hamiltonian, despite employing a similar experimental scheme and setup. In the previous experiment, the states in two paths are $|\varphi_H\rangle = |\psi_H\rangle/\sqrt{\langle\psi_H|\psi_H\rangle}$ and $|\varphi_V\rangle = |\psi_V\rangle/\sqrt{\langle\psi_V|\psi_V\rangle}$ respectively ($|\psi_H\rangle = \hat{U}_{PT}|H\rangle$,

$|\psi_V\rangle = \hat{U}_{PT}|V\rangle$). However, in this experiment, we have $|\varphi'_H\rangle = |\psi'_H\rangle/\sqrt{\langle\psi'_H|\psi'_H\rangle}$ and $|\varphi'_V\rangle = |\psi'_V\rangle/\sqrt{\langle\psi'_V|\psi'_V\rangle}$ ($|\psi'_H\rangle = \hat{U}_\kappa|H\rangle$, $|\psi'_V\rangle = \hat{U}_\kappa|V\rangle$). In this experiment, the total evolution operator is given by

$$\begin{aligned}\hat{U}'_{tot} &= \gamma'(|\psi'_H\rangle\langle H|\otimes|a\rangle\langle a| + |\psi'_V\rangle\langle V|\otimes|b\rangle\langle a| + |\psi'^{\perp}_H\rangle\langle V|\otimes|a\rangle\langle b| + |\psi'^{\perp}_V\rangle\langle H|\otimes|b\rangle\langle b|),\\ &= p'|\varphi'_H\rangle\langle H|\otimes|a\rangle\langle a| + q'|\varphi'_V\rangle\langle V|\otimes|b\rangle\langle a| + p'|\varphi'^{\perp}_H\rangle\langle V|\otimes|a\rangle\langle b| + q'|\varphi'^{\perp}_V\rangle\langle H|\otimes|b\rangle\langle b|,\end{aligned} \tag{S52}$$

where $p'^2/q'^2 = \langle\psi'_H|\psi'_H\rangle/\langle\psi'_V|\psi'_V\rangle$ and $\gamma' = p'/\sqrt{\langle\psi'_H|\psi'_H\rangle} = q'/\sqrt{\langle\psi'_V|\psi'_V\rangle}$. By performing a projective operator $\hat{P} = I\otimes(|a\rangle + |b\rangle)(\langle a| + \langle b|)/2$, we obtain non-unitary evolution operator on probe qubit as follows

$$\hat{U}'_\kappa = (p'|\varphi'_H\rangle\langle H| + q'|\varphi'_V\rangle\langle V|)/\sqrt{2} = \gamma'(|\psi'_H\rangle\langle H| + |\psi'_V\rangle\langle V|) = \gamma'\hat{U}_\kappa, \tag{S53}$$

where $|\varphi'_H\rangle$ and $|\varphi'_V\rangle$ are controlled by the angle of H5, Q1, H6 and Q2. For each time point, we adjust these wave plates to ensure that the evolution operator is $\hat{U}'_\kappa(t)$. To illustrate the distinctions between the two experiments, we present the angle configurations of H6 and Q2 at the same time points in both experiments (here, the probe state is $|0\rangle$, and it does not pass through H5 and Q1), as shown in Table. S6.

We input the probe state $|\psi_0\rangle = |0\rangle$ and perform $n = 1000 \sim 1200$ measurements $\hat{A} = |0\rangle\langle 0|$ on output states for varying time t, where $\hat{A}$ is the corresponding optimal measurement for $|\psi_0\rangle$ according to the condition for optimal measurements Eq. (S35),

$$|f\rangle = \frac{-i[2t\sqrt{\kappa} - \sin(2t\sqrt{\kappa})]}{2\kappa\sin(2t\sqrt{\kappa})}|g\rangle. \tag{S54}$$

The actual value of parameter is $\kappa = 2$. For each time point, we repeat maximum likelihood estimation for 1000 times to obtain the distributions of the estimator $\hat{\kappa}$. As shown in Fig. S10 , we present the estimation precision for varying time. The experimental results reveal that the precision follows Heisenberg scaling t which aligns with the theoretical prediction. The exact experimental data is shown in Table. S7.

For this Hamiltonian without specific symmetries, the experimental results also match with the theory, which exhibits the generality of our theory.

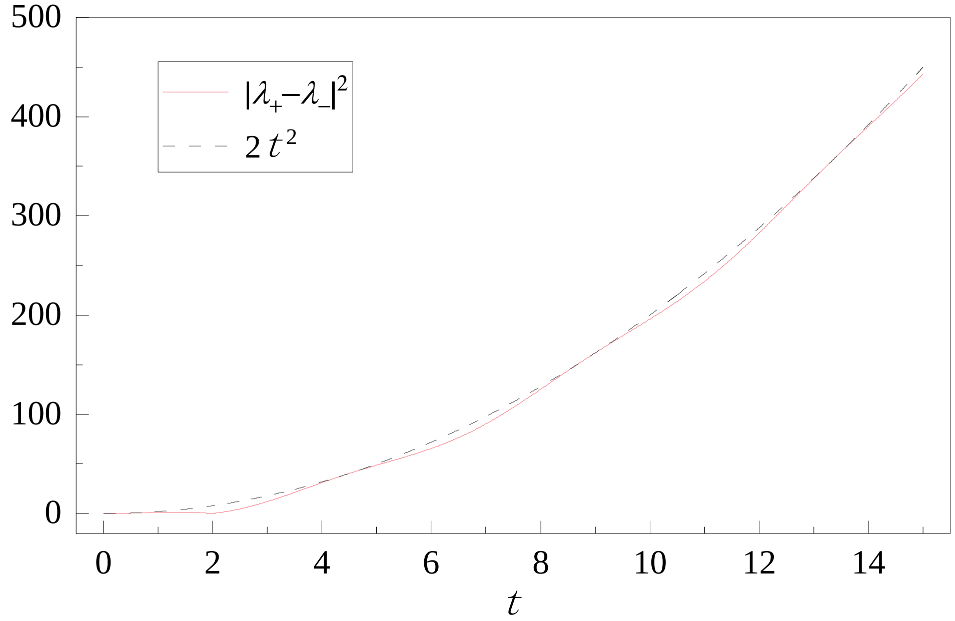

Figure S1: **The evolution of the modulus of difference $|\lambda_+ - \lambda_-|^2$ as function of time t.**

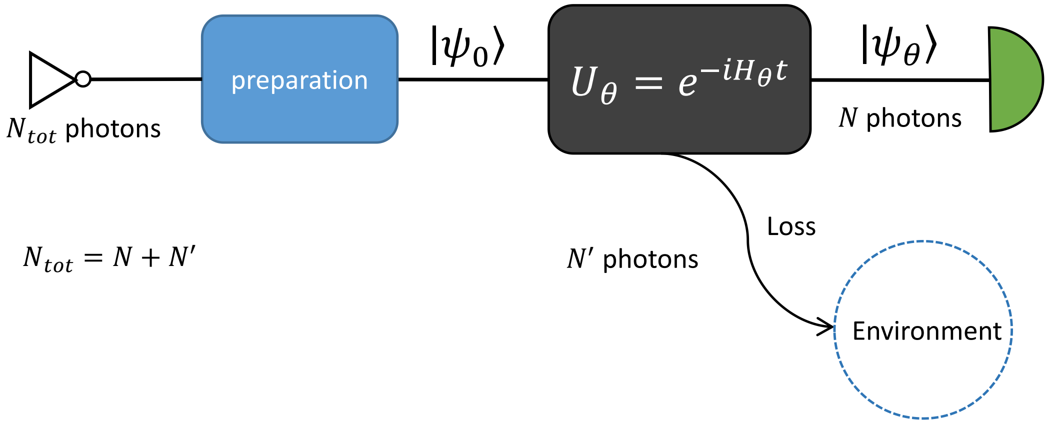

Figure S2: **Simple diagram of quantum estimation in non-Hermitian system.**

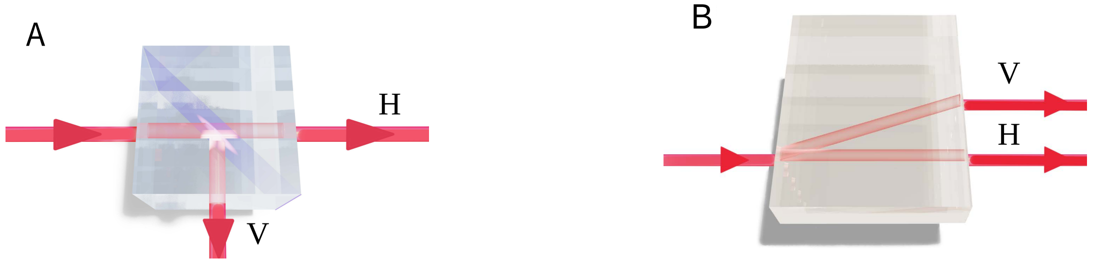

Figure S3: **Schematic illustration of PBS (A) and BD (B).**

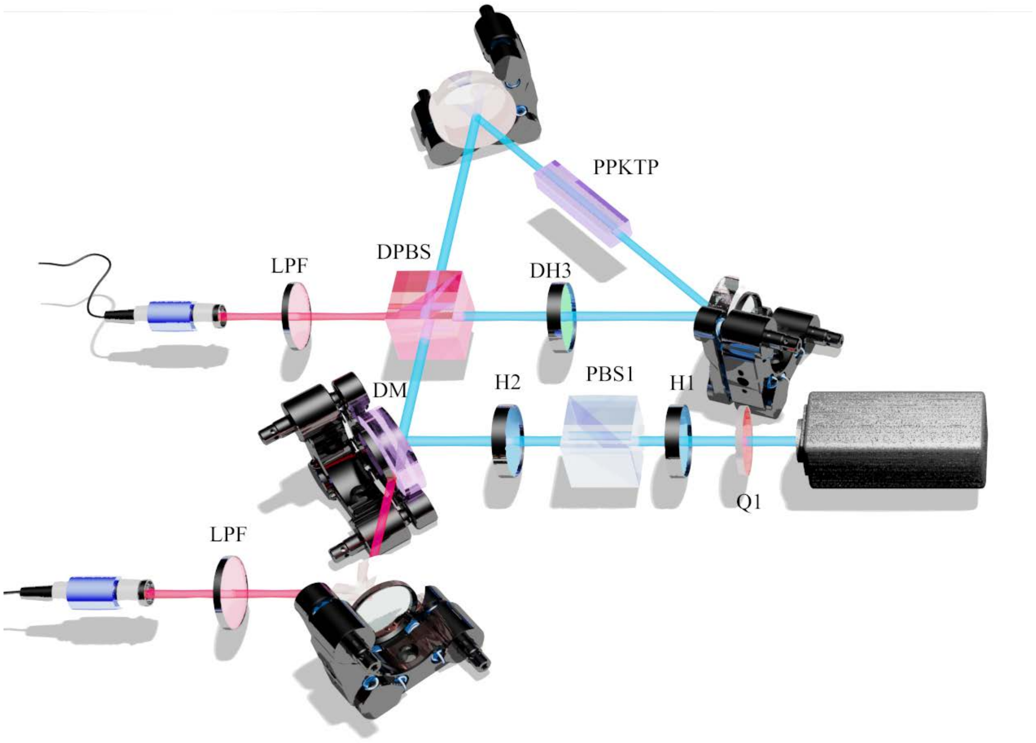

Figure S4: **Schematic illustration of the photon-pair source.**

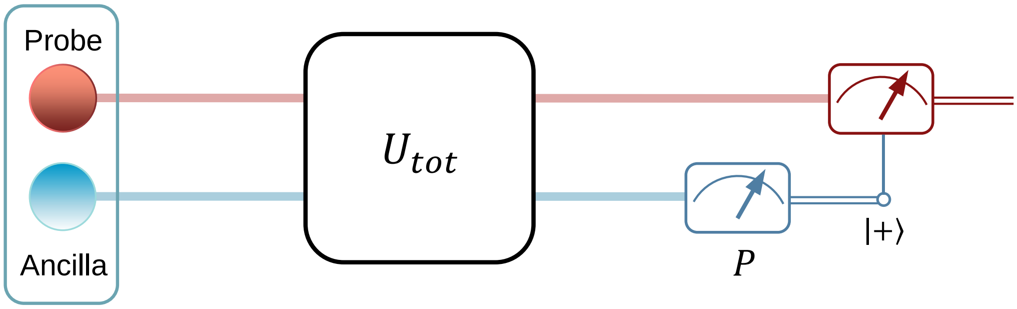

Figure S5: **Post-selected scheme for the non-unitary evolution.** The operator U_{tot} is an evolution with loss process, we effectively obtain the evolution $\hat{U}'_{PT} = F\hat{U}_{PT}$ of the PT-symmetric Hamiltonian $\hat{H}_{PT}$ for probe qubit after post-selection.

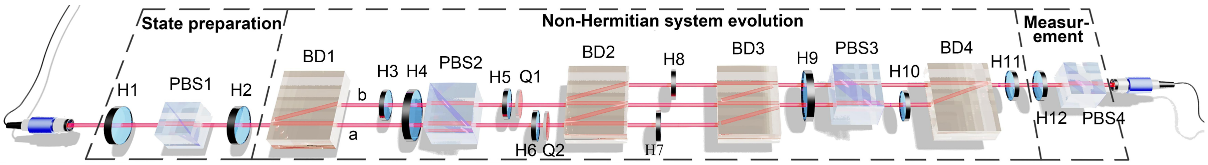

Figure S6: **Schematic illustration of the non-Hermitian system evolution.**

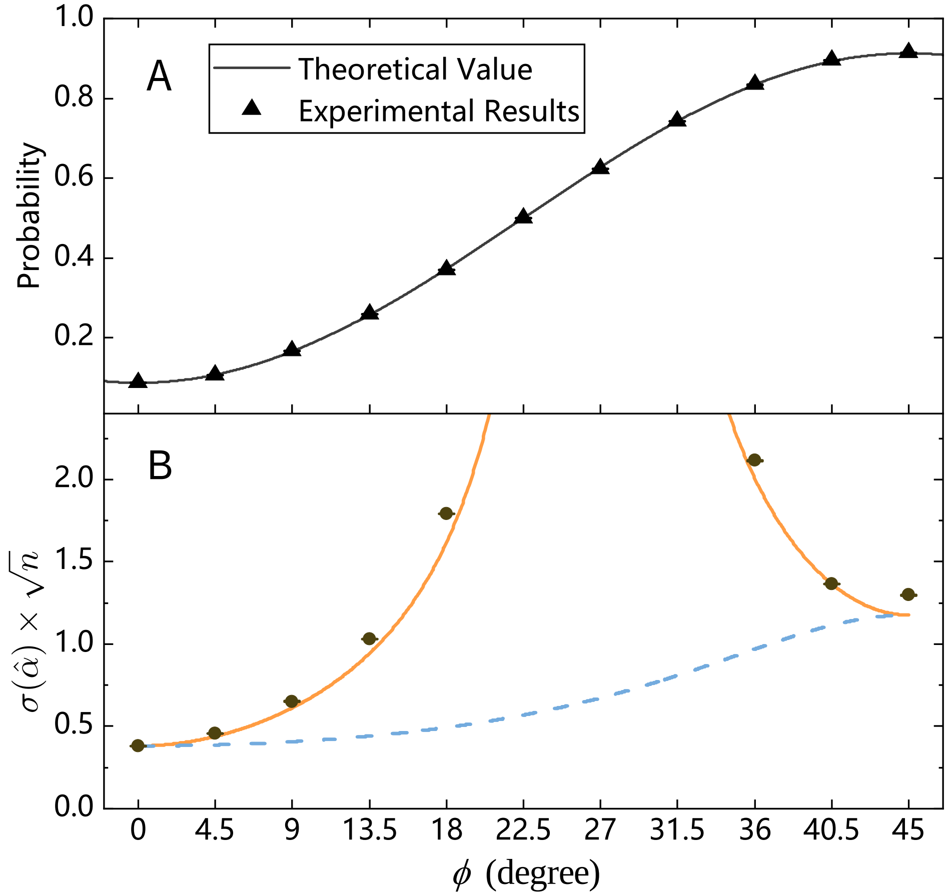

Figure S7: **Estimation precision.** (A) The probabilities of measurement outcomes for different probe states after evolution. We set the measurement as $\hat{A} = |0\rangle\langle 0|$, the probe states $|\psi_0\rangle = \cos 2\phi|0\rangle + \sin 2\phi|1\rangle$ is changed from $\phi = 0°$ to $\phi = 45°$. (B) The standard deviation. The experimental standard deviation (black dots) approximately matches well the theoretical estimation precision (orange solid lines) calculated by the error-propagation formula. The ultimate precision described by QFI (blue dashed lines) is achieved when the probe state is $|0\rangle$ or $|1\rangle$.

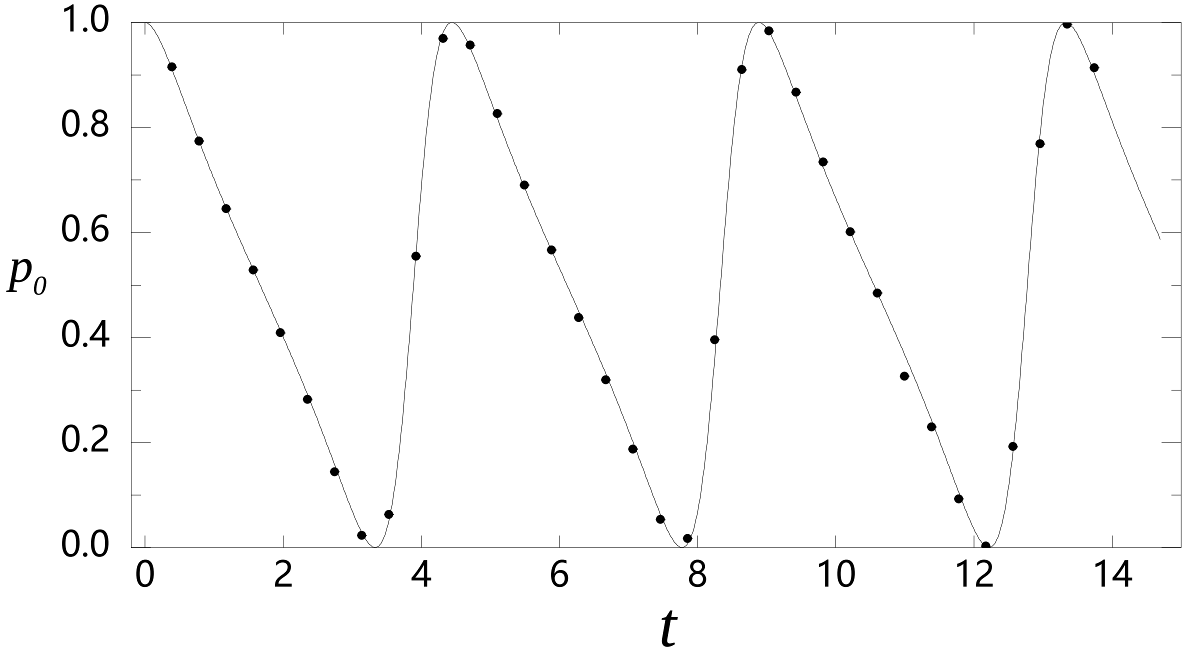

Figure S8: **The probabilities of measurement outcomes for varying t.** The black dots represent the experimentally measured data of P_0 for varying t. And we set $s = 1$, $\alpha = \pi/4$, the measurement performed is $\hat{A} = |0\rangle\langle 0|$ and the probe state is $|\psi_0\rangle = |0\rangle$. The black solid line is the theoretical value of $p_0 = \langle\varphi|\hat{A}|\varphi\rangle$, the data points match well with the theoretical curve.

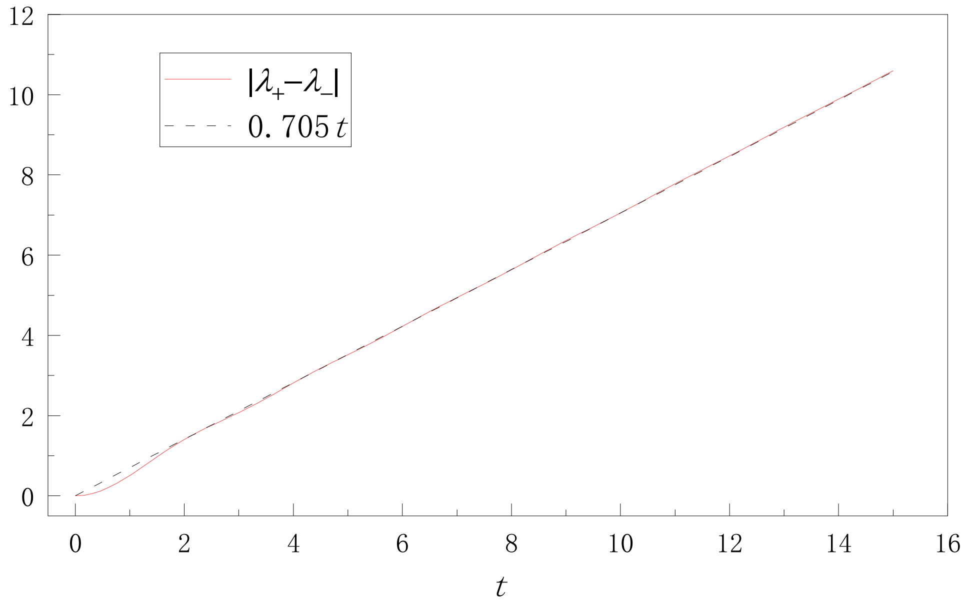

Figure S9: **The evolution of the modulus of difference $|\lambda_+ - \lambda_-|$ as function of time t.**

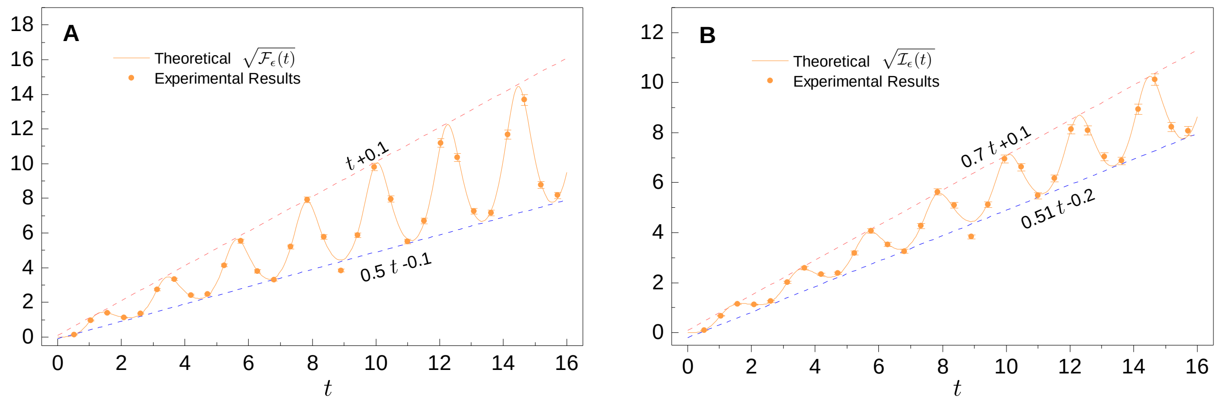

Figure S10: **QFI for varying time t.** The probe state is set as $|0\rangle$, the measurement performed is $\hat{A}$, and the condition for optimal measurements is satisfied. The practical value of κ we set is 2. (A) The square root of QFI, the orange dots are the experimental data and the orange solid line is the theoretical value of $\sqrt{\mathcal{F}_\kappa(t)}$. (B) The QFI multiplied by normalized coefficient K_κ.

Table S1: The measurement results for different probe states.

ϕ	**Experiment p_0**	**Theoretical p_0**
$0°$	0.087393 ± 0.000051	0.0872
$4.5°$	0.106596 ± 0.000053	0.1074
$9°$	0.167888 ± 0.000065	0.1660
$13.5°$	0.258250 ± 0.000075	0.2573
$18°$	0.370123 ± 0.000087	0.3724
$22.5°$	0.499996 ± 0.000089	0.5000
$27°$	0.622651 ± 0.000086	0.6276
$31.5°$	0.742903 ± 0.000078	0.7427
$36°$	0.833638 ± 0.000064	0.8340
$40.5°$	0.896258 ± 0.000050	0.8926
$45°$	0.913169 ± 0.000045	0.9128

Table S2: The standard deviations of $\hat{\alpha}$ for different probe states.

ϕ	Experiment $\sigma(\hat{\alpha})$	Theoretical $\sigma(\hat{\alpha})$	$1/\sqrt{\mathcal{F}_\alpha}$
$0°$	0.3823 ± 0.0001	0.3813	0.3813
$4.5°$	0.4556 ± 0.0002	0.4391	0.3875
$9°$	0.6520 ± 0.0002	0.6128	0.4068
$13.5°$	1.0282 ± 0.0004	0.9425	0.4409
$18°$	1.7911 ± 0.0006	1.6165	0.4934
$22.5°$	——	3.6517	0.5689
$27°$	——	133.1113	0.6732
$31.5°$	——	3.9067	0.8094
$36°$	2.1140 ± 0.0008	2.0078	0.9690
$40.5°$	1.3645 ± 0.0005	1.3661	1.1142
$45°$	1.2968 ± 0.0005	1.1763	1.1763

Table S3: The standard deviations of $\hat{s}$ for different times.

t	**Experiment** $1/\sigma(\hat{s})$	$\sqrt{\mathcal{F}_s(t)}$	$E[\hat{s}]$	**error**(s)
$\pi/8$	0.4612 ± 0.0146	0.4682	0.9644 ± 0.0018	3.5640%
$2\pi/8$	0.6449 ± 0.0204	0.6406	0.9968 ± 0.0012	0.3220%
$3\pi/8$	0.7849 ± 0.0248	0.7624	1.0010 ± 0.0010	-0.0997%
$4\pi/8$	0.9083 ± 0.0287	0.9236	0.9982 ± 0.0009	0.1785%
$5\pi/8$	1.1755 ± 0.0372	1.1933	1.0057 ± 0.0007	-0.5673%
$6\pi/8$	1.7708 ± 0.0560	1.6875	1.0104 ± 0.0005	-1.0438%
$7\pi/8$	2.7532 ± 0.0871	2.6743	1.0123 ± 0.0003	-1.2325%
$8\pi/8$	4.9603 ± 0.1569	4.8245	1.0064 ± 0.0002	-0.6429
$9\pi/8$	9.1263 ± 0.2887	9.3179	1.0043 ± 0.0001	-0.4260%
$10\pi/8$	13.1820 ± 0.4171	13.3574	0.9981 ± 0.0001	0.1869%

Table S4: The standard deviations of $\hat{\alpha}$ for different times.

t	Experiment $1/\sigma(\hat{\alpha})$	$\sqrt{\mathcal{F}_\alpha(t)}$	$E[\hat{\alpha}]$	**error**(α)
$2\pi/8$	0.4310 ± 0.0136	0.4445	0.7934 ± 0.0019	1.0240%
$3\pi/8$	0.8284 ± 0.0262	0.8080	0.7855 ± 0.0010	0.0170%
$4\pi/8$	1.2346 ± 0.0391	1.2604	0.7872 ± 0.0007	0.2267%
$5\pi/8$	1.8511 ± 0.0586	1.8711	0.7819 ± 0.0004	0.4403%
$6\pi/8$	2.9330 ± 0.0928	2.7872	0.7791 ± 0.0003	0.8009%
$7\pi/8$	4.4336 ± 0.1403	4.3343	0.7778 ± 0.0002	0.9730%
$8\pi/8$	7.3842 ± 0.2937	7.2462	0.7811 ± 0.0001	0.5491%
$9\pi/8$	12.0692 ± 0.3819	12.4451	0.7822 ± 0.0001	0.4091%
$10\pi/8$	15.4574 ± 0.4891	15.5728	0.7870 ± 0.0001	0.2029%

Table S5: **The measurement results for different evolution times.**

t	**Experiment** p_0	**Theoretical** p_0
$\pi/8$	0.9151 ± 0.0005	0.9104
$2\pi/8$	0.7742 ± 0.0007	0.7733
$3\pi/8$	0.6453 ± 0.0008	0.6457
$4\pi/8$	0.5288 ± 0.0009	0.5279
$5\pi/8$	0.4089 ± 0.0009	0.4123
$6\pi/8$	0.2822 ± 0.0007	0.2903
$7\pi/8$	0.1443 ± 0.0006	0.1563
$8\pi/8$	0.0229 ± 0.0003	0.0277
$9\pi/8$	0.0630 ± 0.0004	0.0535
$10\pi/8$	0.5547 ± 0.0008	0.5671

Table S6: The angle configurations of H6 and Q2 for varying times in two experiments.

t		$\pi/2$	π	$3\pi/2$	2π	$5\pi/2$	3π	$7\pi/2$	4π
$\hat{H}_{PT}$	H6	$-21.70°$	$-40.21°$	$83.56°$	$66.00°$	$42.61°$	$-11.00°$	$-26.33°$	$-57.71°$
	Q2	$0°$	$0°$	$0°$	$0°$	$0°$	$0°$	$0°$	$0°$
$\hat{H}_{\kappa}$	H6	$111.45°$	$55.67°$	$-7.91°$	$101.46°$	$40.50°$	$-17.01°$	$93.21°$	$26.39°$
	Q2	$0°$	$0°$	$0°$	$0°$	$0°$	$0°$	$0°$	$0°$

Table S7: The standard deviations of $\hat{\kappa}$ for different times.

t	Experiment $1/\sigma(\hat{\kappa})$	$\sqrt{\mathcal{F}_{\kappa}(t)}$	$E[\hat{\kappa}]$	**error(κ)**
$\pi/6$	0.1139 ± 0.0025	0.1110	1.9841 ± 0.2777	-0.7938%
$2\pi/6$	0.9518 ± 0.0213	0.9762	1.9936 ± 0.0332	-0.3181%
$3\pi/6$	1.3815 ± 0.0310	1.3985	1.9955 ± 0.0229	-0.2260%
$4\pi/6$	1.1233 ± 0.0251	1.1274	1.9933 ± 0.0282	-0.3328%
$5\pi/6$	1.3494 ± 0.0302	1.3392	2.0172 ± 0.0235	0.8581%
$6\pi/6$	2.7372 ± 0.0612	2.7642	2.0038 ± 0.0116	0.1882%
$7\pi/6$	3.3269 ± 0.0744	3.2765	1.9905 ± 0.0095	-0.4738%
$8\pi/6$	2.4127 ± 0.0540	2.3565	1.9863 ± 0.0131	-0.6873%
$9\pi/6$	2.4628 ± 0.0551	2.4002	1.9945 ± 0.0128	-0.2758%
$10\pi/6$	4.1355 ± 0.0925	4.1726	1.9983 ± 0.0077	-0.0841%

REFERENCES AND NOTES

1. C. M. Bender, S. Boettcher, Real spectra in non-hermitian Hamiltonians having $\mathcal{PT}$ symmetry. *Phys. Rev. Lett.* **80**, 5243 (1998).
2. C. M. Bender, D. C. Brody, H. F. Jones, Complex extension of quantum mechanics. *Phys. Rev. Lett.* **89**, 270401 (2004).
3. L. Feng, Z. J. Wong, R.-M. Ma, Y. Wang, X. Zhang, Single-mode laser by parity-time symmetry breaking. *Science* **346**, 972–975 (2014).
4. H. Hodaei, M.-A. Miri, M. Heinrich, D. N. Christodoulides, M. Khajavikhan, Parity-time-symmetric microring lasers. *Science* **346**, 975–978 (2014).
5. P. Miao, Z. Zhang, J. Sun, W. Walasik, S. Longhi, N. M. Litchinitser, L. Feng, Orbital angular momentum microlaser. *Science* **353**, 464–467 (2016).
6. S. Longhi, $\mathcal{PT}$-symmetric laser absorber. *Phys. Rev. A* **82**, 031801 (2010).
7. Y. D. Chong, L. Ge, A. D. Stone, $\mathcal{PT}$-symmetry breaking and laser-absorber modes in optical scattering systems. *Phys. Rev. Lett.* **106**, 093902 (2011).
8. Y. Sun, W. Tan, H.-q. Li, J. Li, H. Chen, Experimental demonstration of a coherent perfect absorber with PT phase transition. *Phys. Rev. Lett.* **112**, 143903 (2014).
9. J. Doppler, A. A. Mailybaev, J. Böhm, U. Kuhl, A. Girschik, F. Libisch, T. J. Milburn, P. Rabl, N. Moiseyev, S. Rotter, Dynamically encircling an exceptional point for asymmetric mode switching. *Nature* **537**, 76–79 (2016).
10. H. Xu, D. Mason, L. Jiang, J. G. E. Harris, Topological energy transfer in an optomechanical system with exceptional points. *Nature* **537**, 80–83 (2016).
11. M. Kang, F. Liu, J. Li, Effective spontaneous $\mathcal{PT}$-symmetry breaking in hybridized metamaterials. *Phys. Rev. A* **87**, 053824 (2013).
12. M. Kang, J. Chen, Y. D. Chong, Chiral exceptional points in metasurfaces. *Phys. Rev. A* **94**, 033834 (2016).
13. S. Xiao, J. Gear, S. Rotter, J. Li, Effective PT-symmetric metasurfaces for subwavelength amplified sensing. *New J. Phys.* **18**, 085004 (2016).
14. R. Fleury, D. L. Sounas, A. Alù, Negative refraction and planar focusing based on parity-time symmetric metasurfaces. *Phys. Rev. Lett.* **113**, 023903 (2014).
15. B. Peng, Ş. K. Özdemir, F. Lei, F. Monifi, M. Gianfreda, G. L. Long, S. Fan, F. Nori, C. M. Bender, L. Yang, Parity-time-symmetric whispering-gallery microcavities. *Nat. Phys.* **10**, 394–398 (2014).

16. L. Feng, M. Ayache, J. Huang, Y.-L. Xu, M.-H. Lu, Y.-F. Chen, Y. Fainman, A. Scherer, Nonreciprocal light propagation in a silicon photonic circuit. *Science* **333**, 729–733 (2011).
17. J. Wiersig, Enhancing the sensitivity of frequency and energy splitting detection by using exceptional points: Application to microcavity sensors for single-particle detection. *Phys. Rev. Lett.* **112**, 203901 (2014).
18. J. Wiersig, Sensors operating at exceptional points: General theory. *Phys. Rev. A* **93**, 033809 (2016).
19. Z.-P. Liu, J. Zhang, Ş. K. Özdemir, B. Peng, H. Jing, X.-Y. Lü, C.-W. Li, L. Yang, F. Nori, Y.-x. Liu, Metrology with $\mathcal{PT}$-symmetric cavities: Enhanced sensitivity near the $\mathcal{PT}$-phase transition. *Phys. Rev. Lett.* **117**, 110802 (2016).
20. W. Chen, Ş. K. Özdemir, G. Zhao, J. Wiersig, L. Yang, Exceptional points enhance sensing in an optical microcavity. *Nature* **548**, 192–196 (2017).
21. H. Hodaei, A. U. Hassan, S. Wittek, H. Garcia-Gracia, R. El-Ganainy, D. N. Christodoulides, M. Khajavikhan, Enhanced sensitivity at higher-order exceptional points. *Nature* **548**, 187–191 (2017).
22. H.-K. Lau, A. A. Clerk, Fundamental limits and non-reciprocal approaches in non-Hermitian quantum sensing. *Nat. Commun.* **9**, 4320 (2018).
23. M. Zhang, W. Sweeney, C. W. Hsu, L. Yang, A. D. Stone, L. Jiang, Quantum noise theory of exceptional point amplifying sensors. *Phys. Rev. Lett.* **123**, 180501 (2019).
24. C. Chen, L. Jin, R.-B. Liu, Sensitivity of parameter estimation near the exceptional point of a non-Hermitian system. *New J. Phys.* **21** 083002 (2019).
25. J. Wang, D. Mukhopadhyay, G. S. Agarwal, Quantum Fisher information perspective on sensing in anti-PT symmetric systems. *Phys. Rev. Res.* **4**, 013131 (2022).
26. J.-S. Tang, Y.-T. Wang, S. Yu, D.-Y. He, J.-S. Xu, B.-H. Liu, G. Chen, Y.-N. Sun, K. Sun, Y.-J. Han, C.-F. Li, G.-C. Guo, Experimental investigation of the no-signalling principle in parity-time symmetric theory using an open quantum system. *Nat. Photonics* **10**, 642–646 (2016).
27. Q. Li, C.-J. Zhang, Z.-D. Cheng, W.-Z. Liu, J.-F. Wang, F.-F. Yan, Z.-H. Lin, Y. Xiao, K. Sun, Y.-T. Wang, J.-S. Tang, J.-S. Xu, C.-F. Li, G.-C. Guo, Experimental simulation of anti-parity-time symmetric Lorentz dynamics. *Optica* **6**, 67–71 (2019).
28. Y.-T. Wang, Z.-P. Li, S. Yu, Z.-J. Ke, W. Liu, Y. Meng, Y.-Z. Yang, J.-S. Tang, C.-F. Li, G.-C. Guo, Experimental investigation of state distinguishability in parity-time symmetric quantum dynamics. *Phys. Rev. Lett.* **124**, 230402 (2020).

29. S. Yu, Y. Meng, J.-S. Tang, X.-Y. Xu, Y.-T. Wang, P. Yin, Z.-J. Ke, W. Liu, Z.-P. Li, Y.-Z. Yang, G. Chen, Y.-J. Han, C.-F. Li, G.-C. Guo, Experimental investigation of quantum $\mathcal{PT}$-enhanced sensor. *Phys. Rev. Lett.* **125**, 240506 (2020).
30. L. Xiao, X. Zhan, Z. H. Bian, K. K. Wang, X. Zhang, X. P. Wang, J. Li, K. Mochizuki, D. Kim, N. Kawakami, W. Yi, H. Obuse, B. C. Sanders, P. Xue, Observation of topological edge states in parity-time-symmetric quantum walks. *Nat. Phys.* **13**, 1117–1123 (2017).
31. L. Xiao, K. Wang, X. Zhan, Z. Bian, K. Kawabata, M. Ueda, W. Yi, P. Xue, Observation of critical phenomena in parity-time-symmetric quantum dynamics. *Phys. Rev. Lett.* **123**, 230401 (2019).
32. J. Li, A. K. Harter, J. Liu, L. de Melo, Y. N. Joglekar, L. Luo, Observation of parity-time symmetry breaking transitions in a dissipative Floquet system of ultracold atoms. *Nat. Commun.* **10**, 855 (2019).
33. Y. Jiang, Y. Mei, Y. Zuo, Y. Zhai, J. Li, J. Wen, S. Du, Anti-parity-time symmetric optical four-wave mixing in cold atoms. *Phys. Rev. Lett.* **123**, 193604 (2019).
34. L. Ding, K. Shi, Q. Zhang, D. Shen, X. Zhang, W. Zhang, Experimental determination of $\mathcal{PT}$-symmetric exceptional points in a single trapped ion. *Phys. Rev. Lett.* **126**, 083604 (2021).
35. W.-C. Wang, Y.-L. Zhou, H.-L. Zhang, J. Zhang, M.-C. Zhang, Y. Xie, C.-W. Wu, T. Chen, B.-Q. Ou, W. Wu, H. Jing, P.-X. Chen, Observation of $\mathcal{PT}$-symmetric quantum coherence in a single-ion system. *Phys. Rev. A* **103**, L020201 (2021).
36. M. Naghiloo, M. Abbasi, Y. N. Joglekar, K. W. Murch, Quantum state tomography across the exceptional point in a single dissipative qubit. *Nat. Phys.* **15**, 1232–1236 (2019).
37. M. Partanen, J. Goetz, K. Y. Tan, K. Kohvakka, V. Sevriuk, R. E. Lake, R. Kokkoniemi, J. Ikonen, D. Hazra, A. Mäkinen, E. Hyyppä, L. Grönberg, V. Vesterinen, M. Silveri, M. Möttönen, Exceptional points in tunable superconducting resonators. *Phys. Rev. B* **100**, 134505 (2019).
38. Y. Wu, W. Liu, J. Geng, X. Song, X. Ye, C.-K. Duan, X. Rong, J. Du, Observation of parity-time symmetry breaking in a single-spin system. *Science* **364**, 878–880 (2019).
39. W. Liu, Y. Wu, C.-K. Duan, X. Rong, J. Du, Dynamically encircling an exceptional point in a real quantum system. *Phys. Rev. Lett.* **126**, 170506 (2021).
40. C. M. Bender, D. C. Brody, H. F. Jones, B. K. Meister, Faster than Hermitian quantum mechanics. *Phys. Rev. Lett.* **98**, 040403 (2007).
41. C. Zheng, L. Hao, G. L. Long, Observation of a fast evolution in a parity-time-symmetric system. *Phil. Trans. A Math. Phys. Eng. Sci.* **371**, 20120053 (2013).

42. J. Li, H. Liu, Z. Wang, X. X. Yi, Enhanced parameter estimation by measurement of non-Hermitian operators. *AAPPS Bull.* **33**, 22 (2023).
43. J. J. Bollinger, W. M. Itano, D. J. Wineland, D. J. Heinzen, Optimal frequency measurements with maximally correlated states. *Phys. Rev. A* **54**, R4649–R4652 (1996).
44. V. Giovannetti, S. Lloyd, L. Maccone, Quantum-enhanced measurements: Beating the standard quantum limit. *Science* **306**,1330–1336(2004).
45. V. Giovannetti, S. Lloyd, L. Maccone, Quantum metrology. *Phys. Rev. Lett.* **96**, 010401 (2006).
46. Q. Liu, Z. Hu, H. Yuan, Y. Yang, Optimal strategies of quantum metrology with a strict hierarchy. *Phys. Rev. Lett.* **130**, 070803 (2023).
47. T. Nagata, R. Okamoto, J. L. O'Brien, K. Sasaki, S. Takeuchi, Beating the standard quantum limit with four-entangled photons. *Science* **316**, 726–729 (2007).
48. G. Y. Xiang, B. L. Higgins, D. W. Berry, H. M. Wiseman, G. J. Pryde, Entanglement-enhanced measurement of a completely unknown optical phase. *Nat. Photonics* **5**, 43–47 (2011).
49. R. Okamoto, H. F. Hofmann, T. Nagata, J. L. O'Brien, K. Sasaki, S. Takeuchi, Beating the standard quantum limit: Phase super-sensitivity of *N*-photon interferometers. *New J. Phys.* **10**, 073033 (2008).
50. B. L. Higgins, D. W. Berry, S. D. Bartlett, H. M. Wiseman, G. J. Pryde, Entanglement-free Heisenberg-limited phase estimation. *Nature* **450**, 393–396 (2007).
51. H. Yuan, C.-H. F. Fung, Optimal feedback scheme and universal time scaling for Hamiltonian parameter estimation. *Phys. Rev. Lett.* **115**, 110401 (2015).
52. D. Braun, G. Adesso, F. Benatti, R. Floreanini, U. Marzolino, M. W. Mitchell, S. Pirandola, Quantum-enhanced measurements without entanglement. *Rev. Mod. Phys.* **90**, 035006 (2018).
53. Z. Hou, R.-J. Wang, J.-F. Tang, H. Yuan, G.-Y. Xiang, C.-F. Li, G.-C. Guo, Control-enhanced sequential scheme for general quantum parameter estimation at the Heisenberg limit. *Phys. Rev. Lett.* **123**, 040501 (2019).
54. S. Zhou, M. Zhang, J. Preskill, L. Jiang, Achieving the Heisenberg limit in quantum metrology using quantum error correction. *Nat. Commun.* **9**, 78 (2018).
55. R. Demkowicz-Dobrzański, J. Czajkowski, P. Sekatski, Adaptive quantum metrology under general Markovian noise. *Phys. Rev. X*, **7**, 041009 (2017).
56. C. W. Helstrom, *Quantum Detection and Estimation Theory* (Academic Press, 1976).
57. A. S. Holevo, *Probabilistic and Statistical Aspects of Quantum Theory* (North-Holland, 1982).

58. G. Tóth, I. Apellaniz, Quantum metrology from a quantum information science perspective. *J. Phys. A Math. Theor.* **47**, 42400 (2014).
59. S. L. Braunstein, C. M. Caves, Statistical distance and the geometry of quantum states. *Phys. Rev. Lett.* **72**, 3439–3443 (1994).
60. S. L. Braunstein, C. M. Caves, G. J. Milburn, Generalized uncertainty relations: Theory, examples, and Lorentz invariance. *Ann. Phys.* **247**, 135–173 (1996).
61. S. Pang, T. A. Brun, Quantum metrology for a general Hamiltonian parameter. *Phys. Rev. A* **90**, 022117 (2014).
62. U. Günther, B. F. Samsonov, Naimark-dilated $\mathcal{PT}$-symmetric brachistochrone. *Phys. Rev. Lett.* **101**, 230404 (2008).
63. X. Yu, C. Zhang, Quantum parameter estimation of non-Hermitian systems with optimal measurements. *Phys. Rev. A* **108**, 022215 (2023).
64. D. C. Brody, E.-M. Graefe, Mixed-state evolution in the presence of gain and loss. *Phys. Rev. Lett.* **109**, 230405 (2012).
65. B. Yurke, S. L. McCall, J. R. Klauder, SU(2) and SU(1,1) interferometers. *Phys. Rev. A* **33**, 4033–4054 (1986).
66. S. F. Huelga, C. Macchiavello, T. Pellizzari, A. K. Ekert, M. B. Plenio, J. I. Cirac, Improvement of frequency standards with quantum entanglement. *Phys. Rev. Lett.* **79**, 3865–3868 (1997).
67. A. K. Pati, U. Singh, U. Sinha, Measuring non-Hermitian operators via weak values. *Phys. Rev. A* **92**, 052120 (2015).
68. M. J. W. Hall, A. K. Pati, J. Wu, Products of weak values: Uncertainty relations, complementarity, and incompatibility. *Phys. Rev. A* **93**, 052118 (2016).
69. D. Mondal, S. Bagchi, A. K. Pati, Tighter uncertainty and reverse uncertainty relations. *Phys. Rev. A* **95**, 052117 (2017).
70. B. Yu, N. Jing, X. Li-Jost, Strong unitary uncertainty relations. *Phys. Rev. A* **100**, 022116 (2019).
71. X. Zhao, C. Zhang, Uncertainty relations of non-hermitian operators: Theory and experimental scheme. *Front. Phys.* **10**, 862868 (2022).
72. G.-L. Long, General quantum interference principle and duality computer. *Commun. Theor. Phys.* **45** 825 (2006).
73. T. Kim, M. Fiorentino, F. N. C. Wong, Phase-stable source of polarization-entangled photons using a polarization Sagnac interferometer. *Phys. Rev. A* **73**, 012316 (2006).

74. R. Demkowicz-Dobrzański, J. Kołodyński, M. Guçă, The elusive Heisenberg limit in quantum-enhanced metrology. *Nat. Commun.* **3**, 1063 (2012).
75. K. Kawabata, Y. Ashida, M. Ueda, Information retrieval and criticality in parity-time-symmetric systems. *Phys. Rev. Lett.* **119**, 19040 (2017).
76. R. Cheng, Quantum geometric tensor (Fubini-Study metric) in simple quantum system: A pedagogical introduction. arXiv:1012.1337 (2013).
77. C. Li, M. Chen, P. Cappellaro, A geometric perspective: Experimental evaluation of the quantum Cramer-Rao bound. arXiv:2204.13777 (2022).
78. D. Brody, E.-M. Graefe, Information geometry of complex Hamiltonians and exceptional points. *Entropy* **15**, 3361–3378 (2013).
79. S. Ahn, J. A. Fessler, "Standard errors of mean, variance, and standard deviation estimators" (Technical Report, EECS Department, The University of Michigan, 2003), pp. 1–2.
80. R. M. Wilcox, Exponential operators and parameter differentiation in quantum physics. *J. Math. Phys.* **8**, 962–982 (1967)
